# On the evolution of security and privacy in Android health APIs

**DOI:** 10.3389/fdgth.2026.1818536

**Published:** 2026-10-28

**Authors:** Alejandro Pérez-Fuente, M. Mercedes Martínez-González, Javier Bustos-Jiménez

**Affiliations:** 1 Grupo de Investigación en Ingeniería de la Privacidad, Universidad de Valladolid, Valladolid, Spain; 2 NIC Chile Research Labs, Centro NIC Chile, Universidad de Chile, Santiago, Chile

**Keywords:** Android, health, metadata, mobile applications, privacy, security

## Abstract

**Introduction:**

With the rapid proliferation of mobile health technologies and smart devices, safeguarding sensitive personal health data has become critical. Android 14 introduced Health Connect as a centralized platform designed to optimize health data management, grant users granular control over their information, and enable secure, transparent inter-app communication. However, whether Health Connect represents an effective advance over earlier Android solutions remains to be evaluated.

**Methods:**

A structured, technology-agnostic evaluation framework was designed using a threat model grounded in European cybersecurity (NIS2 Directive, Cyber Resilience Act) and privacy (GDPR) standards. Across defined security and privacy criteria, Health Connect was comparatively evaluated against its predecessor solutions (Google Fit and the Android Sensors API) examining storage integration, encryption, operational models and permission granularity alongside empirical verification using the OWASP Mobile Application Security Testing Guide (MASTG).

**Results:**

Health Connect provides stronger native architectural support for security (availability, integrity and authenticity) and privacy (data minimisation, user consent management, transparency, accuracy and storage limitation) compared to the general-permission, cloud-dependent or developer-dependent models of Google Fit and the Android Sensors API. However, security support for confidentiality remains partial due to an empirical failure in runtime/database-level encryption within its local sandbox.

**Discussion:**

Health Connect marks a significant step forward in Android health data protection, largely driven by fine-grained permissions and local container isolation. Nevertheless, the lack of database-level runtime encryption and sparse technical documentation represent key shortcomings. Addressing these issues and improving implementation transparency are essential to consolidate user and developer trust across the digital health ecosystem.

## 1 Introduction

Security and privacy in health apps are issues of growing importance (1). With the increased use of mobile devices and technology platforms for health monitoring and management, users are constantly sharing sensitive data, such as personal medical and biometric information (2). This collection and constant sharing of health information between different applications presents significant risks, as if not adequately protected, this data could be exposed to misuse. Therefore, developing effective protection and access control mechanisms is crucial to safeguard the integrity of systems where health data is exchanged, and to avoid potential security and privacy breaches that could negatively impact on users’ rights to health privacy.

In this context, Android, the most widely used operating system on mobile devices (3), plays a crucial role in the health app ecosystem. Both wearables and health apps connect to and run on Android. Ultimately, therefore, the security of sensitive health data depends on how secure this operating system is. The growing concern about security and privacy on mobile devices lies, in part, in the difficulty of protecting personal data on an app-specific basis, which exposes users. To improve user security and privacy, Android has evolved the way it manages health data, from systems that only allowed raw access to all health data, to granular management systems where users control which specific data they want to share with which applications. This has been achieved through the introduction of Health Connect, announced at Google I/O in 2022 and launched with Android 14 in October 2023.

Figure 1 illustrates the health data flow within Android 14 mobile ecosystem. In a typical scenario, such as fitness tracking, the health app collects data from various sources, including embedded device sensors and external wearables, and relies on a health data management system (e.g., Android Health APIs) to store and share this information. For the aim of this analysis, the General Data Protection Regulation (GDPR) (4) distinction between *Data Controller* (determines the purpose and means of the processing of personal data) and *Data Processor* (process personal data on behalf of the controller) has been adapted to the mobile applications interactions with APIs they use to manage data. In the interaction between mobile applications and health APIs, the applications play the role of *controller*, while the APIs are *processors* of health data on behalf of health apps. According to the CNIL (5), the processor *“must assist the controller in order to ensure that the rights of individuals are respected*” and *“must take technical and organisational measures to enable exercise of rights*” (p. 48). Consequently, the robustness of the entire privacy and security chain also depends on the processor’s ability to provide such mechanisms. This critical dependency justifies why this study focuses on the analysis of Android Health APIs, as their capacity to simplify or hinder compliance is a determining factor for the overall security and privacy of the user’s health information.

**Figure 1:**
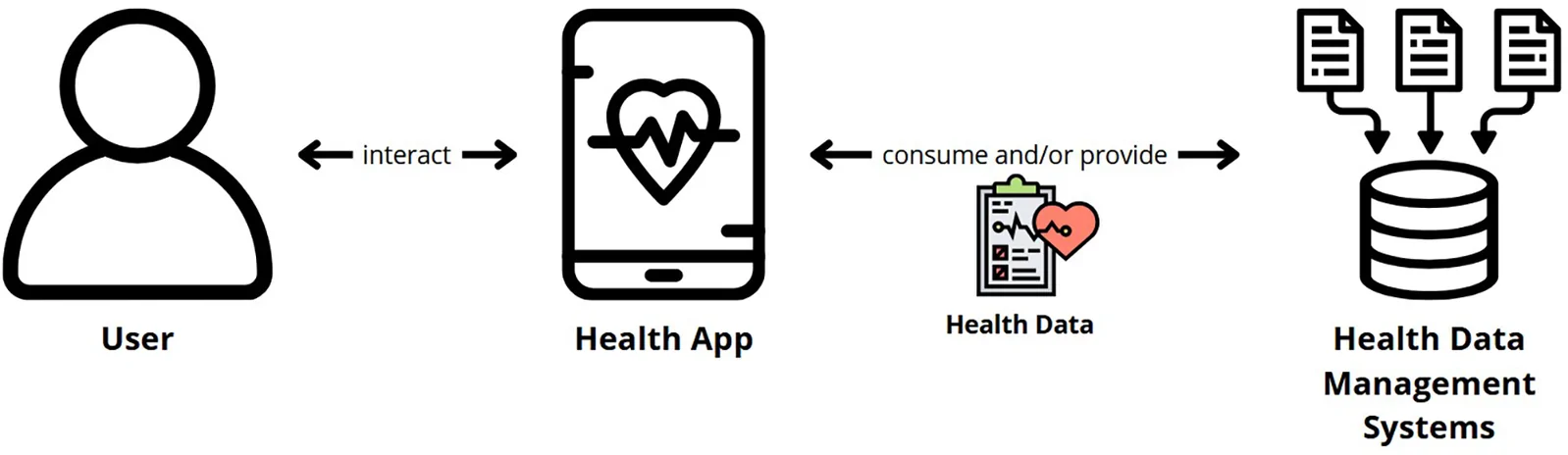
Health data flow in Android 14.

Thus, this study is motivated by the following Research Question: Does Health Connect improve the security and privacy of health data over the solutions that precede it? Furthermore, if so, does it improve both characteristics or only one of them? To answer these questions, we propose a structured methodology to assess the impact of Health Connect on the security and privacy of health information in Android 14, emphasising its implications compared to previous solutions. Notably, this methodology allows for the independent evaluation of both security and privacy aspects. It includes the definition of the assessment framework: materials used, criteria and features evaluated, API selection, and the steps to be performed to answer our research question.

In the first phase, a threat model based on the health data flow shown in Figure 1 is defined, and the resulting privacy and security criteria to guide the analysis are established. In the second phase, the health-related APIs that will be compared are selected. To inform this selection, a study of the evolution of Android with respect to health applications is carried out, allowing the identification of historically used APIs that may still be operational and coexist with Health Connect. As far as we know, none of the existing methodologies used to analyse health apps has adopted a comparative approach involving other technical solutions historically provided by the Android operating system for health data management. This approach ensures a relevant and contextualised comparison. Finally, in the third and fourth phases, the comparative analysis of the selected APIs is performed based on the established criteria in the first phase.

The rest of the article is organised as follows. In Section 1, the state of the art on resource and sensitive data management in Android is reviewed. In Section 2, previous studies related to health data security and privacy, as well as secure storage, encryption and applicable regulations are discussed. In Section 3, the methodology used for the comparative analysis is presented and applied. The changes introduced by Health Connect to data protection and access management mechanisms are examined, highlighting their implications for user privacy and system security in comparison with previous solutions. In Section 4, the results associated with the analysis performed are discussed. In Section 5, the conclusions are presented. Finally, in Section 6, possible lines of future work are proposed.

## 2 Related work

### 2.1 Evolution of security and privacy in android

In 2008, Android introduced a permission system to control application access to device resources and data. At that time, Android only utilised a single type known as *install-time permissions*. These permissions are granted at the exact moment the application is installed. Later, in 2015, with the release of Android 6, the operating system introduced a second type: *runtime permissions*. Unlike install-time permissions, these can be granted or revoked while the app is running. To present these to the user, Android organises runtime permissions into logical groups (6). However, until that point, this model suffered from a serious flaw: a lack of granularity (7). This meant that while users had control over their data, it was a coarse-grained form of control; they could only grant or revoke access to broad resource categories, such as external storage or the gallery.

Ten years later, in 2018, starting with Android 9, the operating system began to focus more actively on user security and privacy by evolving its security model. This was achieved through two parallel approaches: increasing permission granularity and providing new, more private methods for accessing device data (8). To illustrate this, we can look at the evolution of image access. In Android 9 and earlier, an app wanting to access photos simply requested the READ_EXTERNAL_STORAGE permission, and the OS granted access to the entire device storage. In Android 11, the OS introduced specific media permissions (READ_MEDIA_IMAGES, READ_MEDIA_VIDEO, READ_MEDIA_AUDIO), increasing system granularity. Simultaneously, the Photo Picker API was introduced, allowing users to select exactly which images and videos the app could access. Finally, in Android 14, the requirement for apps to request media permissions was removed entirely, relying solely on the Photo Picker API (9).

The evolution of health data on Android has been slower. In 2014, with Android 5, the OS introduced the BODY_SENSORS permission for accessing body sensors. It was not until 2023, with Android 13, that the system provided privacy-aware access to health data via Health Connect.^1^ It was initially introduced as a standalone Android application. However, for backward compatibility, it supported devices running Android 9 and later,^2^ requiring users to download the app from the Play Store to work with Health Connect. From Android 14 onward, Health Connect became an integrated OS framework module, eliminating the need for users to install the application separately. Despite this architectural difference, official documentation clarifies that this transition is completely transparent as the framework module retain all the features within the APK.^3^ Across both versions, this framework introduces granular permissions for specific health data and provides an API through which apps must request data. All these changes and guidelines are published on the Android Developers website,^4^ which currently establishes a clear workflow for requesting permissions^5^ : first, attempt to provide functionality without permissions by using the system’s alternative private mechanisms; if that is not possible, request the permission.

### 2.2 Security and privacy regulations

Security from a regulatory perspective varies depending on the geopolitical region. In this regard, we will focus on European security standards, as this is our current jurisdiction. Two of the most significant European regulations are the NIS2 Directive (Network and Information Security 2) (Directive (EU) 2022/2555) (10) and the CRA (Cyber Resilience Act) (Regulation (EU) 2024/2847) (11). The NIS2 Directive defines cybersecurity and the characteristics that render an information system secure: availability, authenticity, integrity, and confidentiality. The CRA Regulation requires developers and distributors of digital products to implement “security by design” and “security by default”. This is achieved through requirements defined in Annex I, which are closely linked to the characteristics established in the NIS2 Directive.

Similarly to security, privacy regulations also depend on geopolitical regions. In the United States, the Health Insurance Portability and Accountability Act (HIPAA) (12) is a federal law specifically designed to protect health data. In contrast, Europe applies the General Data Protection Regulation (GDPR) (4), which is much broader in scope, protecting not only health data but all types of personal data. Furthermore, the GDPR is significantly more stringent regarding privacy protection (13–15). Key principles safeguarding privacy according to normative include data minimisation, transparency, and consent management. The GDPR is enforced by “supervisory authorities”, which are responsible for verifying regulatory compliance, issuing sanctions for infringements, and providing technical guidance to facilitate adherence to the law. The supervisory authorities for Spain and France, the Spanish Data Protection Agency (AEPD) and the National Commission on Informatics and Liberty (CNIL), have each published specific guidelines tailored to mobile application privacy and compliance (5, 16). For instance, the CNIL emphasises the importance of staying aligned with evolving mobile operating systems to ensure robust data minimisation; this dictates that applications should only collect and process data that is strictly essential for their intended functionality.

### 2.3 Security and privacy in mobile health

Research into the security and privacy of mobile health applications (*mHealth*) has followed two distinct paths: the analysis of existing consumer applications and the analysis of health data management. This current section examines security and privacy within consumer mHealth apps, while Section 2.4 examines the health data management systems.

Consumer mobile health applications have been studied on numerous occasions from the perspectives of security and privacy. From a cybersecurity standpoint, research has been conducted through checks and policies (17, 18), as well as the use of secure authentication and signature protocols (19, 20), encryption standards (21, 22), and even the implementation of blockchain (23, 24). Regarding privacy, studies have examined the lack of transparency in data handling (25–27) and the absence of adequate data access management mechanisms (25, 26, 28). Throughout these studies, end-user health applications have been analysed, with security and privacy issues being detected. However, in no case have the health data management systems provided by mobile operating systems been analysed, even though these should provide mechanisms to prevent such problems, as discussed in Section 1.

### 2.4 Data management in mobile health

As outlined in Section 2.1, the Android operating system provides mechanisms for health data management: the Android permission model and Health Connect. Furthermore, some private companies offer solutions such as *Samsung Health* or *Garmin Connect*; these solutions have been analysed from the end-user perspective, but not as health data management systems (29). The literature also contains proposals to mitigate mHealth privacy and security issues through the design of health data management systems. These range from the use of modular architectures that integrate intrusion detection systems, to the design of applications based on configurable blockchain that guarantee the user full ownership and control over their digital health footprint (30, 31). Across all these systems and studies, it is observed that some of the characteristics that significantly condition them are their architecture (modular or decentralised), their deployment nature, whether cloud-based or local, and the user’s capacity to autonomously manage their identity and privacy. Issues which also will guide our analysis.

## 3 Materials and methods

This framework separates the comparison from specific solutions. This allows the methodology to remain applicable to other operating systems and future technologies. Furthermore, the design aligns with current security and privacy regulations. This is essential given the sensitive nature of the data handled by Android health APIs.

### 3.1 Materials

#### 3.1.1 Sources for security and privacy criteria selection

This section outlines the main materials used to define the assessment framework and to obtain information about health APIs. The assessment framework is structured on the security and privacy criteria that should be assessed, and the technical features that support them. Normative documents have been the main source of information for this aim:

1. **Security issue**. Two European Regulations in the cybersecurity area have been used:
  • The NIS2 (Network and Information Security 2) Directive, which establishes a framework for security governance that concerns digital services providers in the European Union. It requires that security risks are correctly managed by these providers. It concerns IT infrastructures (10).
  • The CRA (Cyber Resilience Act). It introduces a regulatory framework for the security of connected products. Its main novelty is that it includes those who develop and distribute digital products as concerned agents, requiring them to apply “security by design” and “security by default” (11).
2. **Privacy issue**. A legal document and several guides coming from Data Protection Supervisors have been used:
  • The main legal document in the European Union concerning privacy, the General Data Protection Regulation (GDPR) (4).
  • A guide for mobile app privacy assessment, from AEPD (*Agencia Española de Protección de Datos*), the Spanish National Privacy Authority, dated 2019 (16).
  • A guide for mobile app developers, from CNIL (*Commission Nationale de l’Informatique et des Libertés*), the French National Authority, dated 2024 (5).

#### 3.1.2 Sources for health API selection

To apply the assessment framework to health APIs, the main information sources used have been the documentation of each API, and the source code, when available^6^ :

- Android sensors API documentation (32). Source code^7^ available from Android Open Source Project (AOSP).
- Google Fit documentation (33). Due to Google Fit being a proprietary solution, its source code is not publicly available.
- Health Connect documentation (34). Source code^8^ available from AOSP.

### 3.2 Methodology

This section presents the proposed methodology for conducting the comparative analysis. Each phase of the methodology is detailed and applied in the following subsections. Figure 2 illustrates the phases of the proposed methodology.

**Figure 2:**
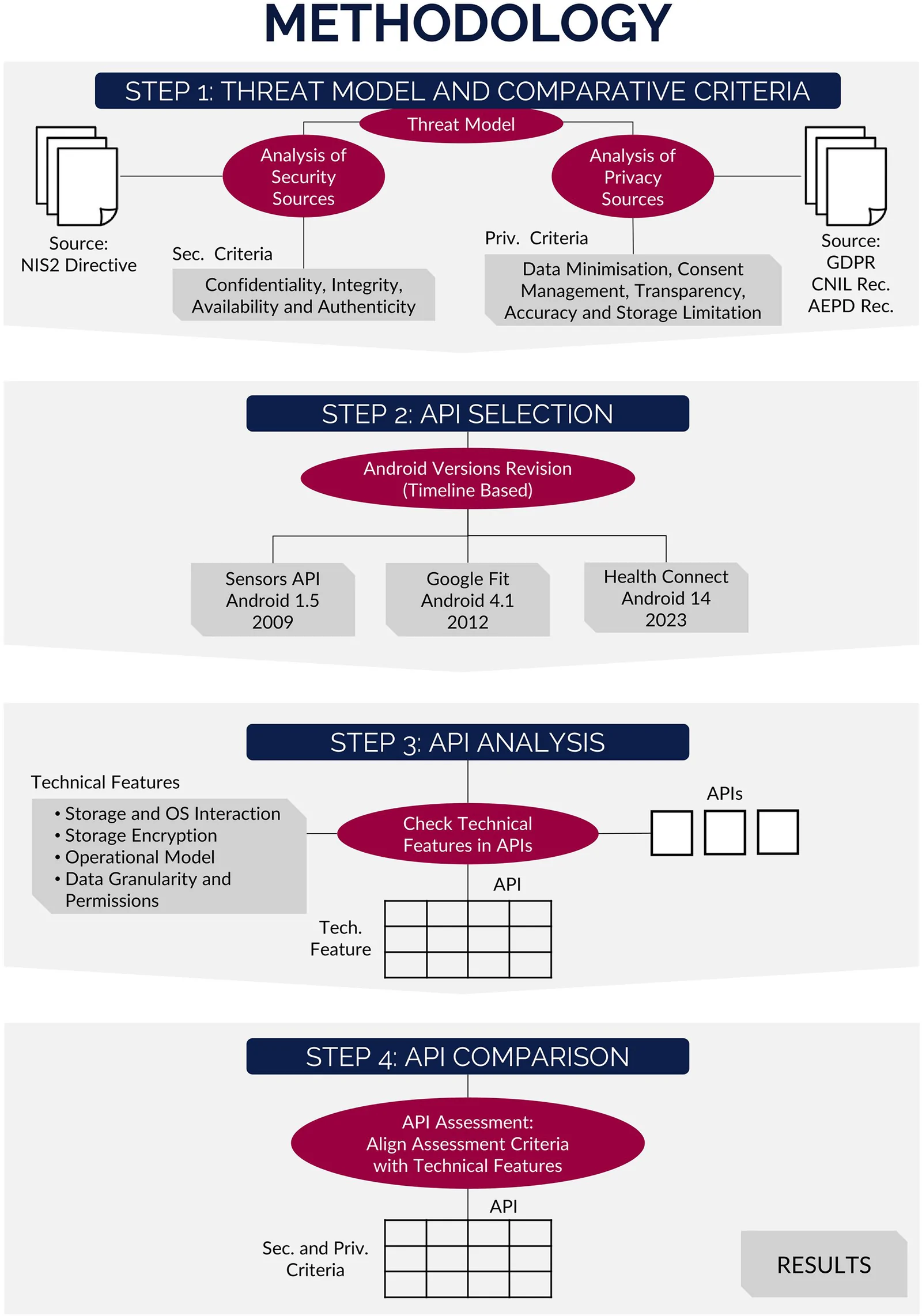
Proposed methodology.

There are four phases. First, the framework is designed: a threat model is defined and the assessment criteria are fixed for each dimension (security, privacy). Next, the current solutions used in the Android ecosystem to manage health data are identified, this is the (health) API selection phase. Following, the analysis is performed: for each API the technical features that can be used to ensure each assessment criteria are checked. Finally, a comparison among the health solutions (APIs) is made.

#### 3.2.1 Phase 1: threat model and comparative criteria

To establish a principled foundation for evaluating health data management APIs, we identify key elements grounded in established threat model methodologies (35–37). Based on the health data flow presented in Figure 1, our model considers the necessary protections at both the device and network levels. The primary *assets* to be safeguarded are the health data and their associated metadata (e.g., acquisition timestamps, origin devices, data sources, and tracking identifiers) stored on the device. We assume that the local device is trustworthy; i.e., that the Android operating system and the inter-process communication (IPC) mechanisms are intact and uncompromised and that no malicious applications are present on the device. While compromised device scenarios (such as local malware) warrant an expanded threat model, they remain outside our scope. Conversely, external entities and transit channels cannot be trusted by default. Within this scope, we define two principal *adversaries*: (i) *network-level eavesdroppers*, who exploit unencrypted channels, insecure communication protocols, or compromised routing to intercept or tamper with health data in transit (1, 38, 39); and (ii) *unauthorised third parties*, who engage in excessive data harvesting, user profiling, or secondary processing without valid user consent (25, 38, 40).

To withstand these adversaries, a health data management solution must provide mechanisms to enforce strict security and privacy guarantees. Specifically, network-level eavesdroppers are countered by ensuring data *confidentiality* and *integrity*, while unauthorized third parties are mitigated through *data minimisation*, explicit *consent management*, and granular *transparency*. The architectural features each API uses to implement these defenses are audited in Phase 3 (see Section 3.2.3). To structure this evaluation, we define the following security and privacy criteria. These are technology-agnostic, ensuring the methodology remains applicable to other operating systems or future solutions:

1. **Security Criteria**. For the selection of security criteria, the European cybersecurity regulations that best apply to mobile devices and applications have been taken into account. The NIS2 Directive establishes security requirements to ensure security resilience of network and information systems, emphasizing *risk assessment*, which concern health sector (of high criticality according Annex I). The CRA also deals with security requirements, but *security by design* and *security by default* are its main focuses. That is, those in charge of the design of digital products, such as health applications, are the main concerned by CRA:
  • *Confidentiality*: Ensures that data are accessible only to those with the appropriate authorisation, preventing leaks or unauthorised access. Part of CRA Requirement (e).
  • *Integrity*: Ensures that information remains accurate and free from unauthorised modifications, preserving its truthfulness and consistency. This is especially crucial during data transmission. Part of CRA Requirement (f).
  • *Availability*: Ensures that both systems and data are available to authorised users when required, preventing disruptions or service outages. Part of CRA Requirement (h).
  • *Authenticity*: Verifies the identity of users and the origin of the information, establishing a solid foundation of trust. Part of CRA Requirement (d).
  While Annex I Part I of the CRA lists broad security requirements, this study focuses exclusively on the APIs’ verifiable technical capabilities. Other requirements are excluded for two reasons: data protection is already covered by the GDPR, and procedural obligations (e.g., patch management) depend on the developer’s maintenance policies rather than the intrinsic system architecture. Centering the analysis purely on architectural design ensures the findings remain applicable regardless of a manufacturer’s update cycles or operational maturity, placing those administrative factors outside our scope.
2. **Privacy Criteria**. Articles 5 (*Principles relating to processing of personal data*) and 6 (*Lawfulness of processing*) of GDPR provide the principles that should be respected when treating personal data. Principles related to verifiable technical features have been considered, that is, those that can be directly supported or facilitated by the health data management APIs. For example, by providing technical features that allow independent access for each type of data, the *data minimisation* principle can be accomplished by the user directly. However, principles such as *purpose limitation* and *accountability* are excluded, as these are more related with the governance policies of the *data controller* (i.e., the end-user app) than to the technical features of the health data management APIs. Consequently, the selected criteria are:
  • *Data Minimisation*: This means that only the data strictly necessary for the intended purpose should be collected, avoiding any excessive processing. For instance, an app that tracks only daily steps should not access the blood pressure sensor.
  • *Consent*: An expression of Lawfulness of processing (art. 6, GDPR). It ensures that data subjects are free to consent and revoke consent in an effective manner. That is, digital products provide the means for their users to consent the use of their data, and revoke consent when they prefer. For health apps, their users should be able to consent, or revoke consent, to use their health data.
  • *Transparency*: Ensures that users are fully informed about the collection, processing, and intended use of their personal data.
  • *Accuracy*: Requires that data remains correct and up to date, so that the rectification or deletion of incorrect information.
  • *Storage Limitation*: Restricts the retention of personal data to the duration strictly necessary for the purposes for which it is processed.${}^{13}$

To assess the degree to which each API satisfies these security and privacy criteria against the defined threat model, a four-tier evaluation scale is established:

- *Native support*: The API neutralises the threat architecturally or automatically, without relying on developer interventions or external configurations.
- *Partial support*: The API provides built-in safeguards or mechanisms, but they do not cover the threat completely or they present verifiable gaps.
- *Developer-dependent*: The API does not protect the data by default, delegating the entire responsibility of implementing safeguards (such as encryption or access restrictions) to the application developer.
- *Not supported*: The API fundamentally lacks the tools, architecture, or mechanisms necessary to satisfy the criterion.

#### 3.2.2 Phase 2: health APIs selection for comparative analysis

During this phase, the APIs for comparative analysis are selected. A historical review of the Android ecosystem was made, identifying the primary frameworks for health data management. Only the native evolution of the Android operating system has been considered, proprietary SDKs (e.g., Samsung Health or Fitbit SDK) were excluded despite their prevalence. To identify the specific APIs, we queried “health management” in the official Android documentation^9^ and followed a recursive discovery process. The primary search result was the recently introduced Health Connect. Within the Health Connect API documentation,^10^ a migration guide references Google Fit, identifying it as a predecessor.^11^ In Google Fit documentation, Table 3 collects “*Migration steps from Fit Sensor API*”, where the Sensor API is identified as an earlier iteration. No further native alternatives were found. The three main APIs that currently coexist are examined, encompassing both legacy and modern frameworks. They are listed below in chronological order:

1. *Sensors API*, dated on 2009, which appeared with Android version 1.5.
2. *Google Fit*, dated on 2012, appeared with Android version 4.1.
3. *Health Connect*, dated on 2023, appeared with Android version 14.

#### 3.2.3 Phase 3: health APIs analysis

The purpose of this analysis is to understand how health data is managed with each of the selected health APIs. The CNIL (5) requirement for *data processors* to provide robust technical measures to ensure that individuals’ rights are respected, can be translated into a requirement for APIs to provide such type of measures when they manage health data. By identifying how each API handles health information, we characterized the evaluation criteria for subsequent methodology phases as concrete architectural mechanisms designed to mitigate the network and third party level adversaries defined in our threat model (see Section 3.2.1). Specifically, these technical features are:

- *Storage and OS Interaction*: Where is data stored, and how this storage integrates with the *Android security model*. Health data may be stored within the device’s local storage under the direct management of the operating system or in external storage, where management falls outside the OS’s native security scope. Features like *verification of origin*, related with authenticity, or *direct access to device sensors*, are characteristic of local storage. Thus, this technical feature provides architectural support for *authenticity*, *confidentiality*, *accuracy* and *storage limitation* by integrating access control directly into the native OS container.
- *Storage Encryption*: This assesses the presence of *encrypted storage* (“encryption at rest”). The analysis identifies whether the system implements native encryption and, when possible, specifies the mechanisms and algorithms employed to protect the stored health information. This technical feature is one of the mechanisms recommended by CRA to provide the security criteria *confidentiality*.
- *Operational Model*: This distinguishes between *local operation*, where data management and access occur strictly on the device, and *cloud-based operation*, where data handling is outsourced to external infrastructure. This provides technical support for criteria such as *integrity*, *availability* and *consent*, as an on-device operational model removes external network dependencies and facilitates localized data governance.
- *Data Granularity and Permissions*: This evaluates the ability to manage access permissions for individual data types independently. It compares the use of *general permissions* (broad access) against *granular permissions*, ensuring that granting access to a specific category (e.g., glucose levels) does not inherently grant access to unrelated health metrics (e.g., heart rate). It provides direct architectural mechanisms to support *data minimisation*, *consent* and category-level *transparency* by enabling users to restrict access on a per-metric basis.

To evaluate the implementation effectiveness of these technical features, we established a validation strategy that contrasts official documentation claims with empirical and literature-based evidence. The architectural capabilities outlined in the Health Connect documentation were verified using the OWASP Mobile Application Security Testing Guide (MASTG) framework (41). To address backward compatibility, this evaluation was conducted in a controlled Android 13 environment (API level 33, x86_64 architecture), analysing Health Connect build 2026.02.19.00.release (versionCode 242770, package com.google.android.apps.healthdata). Synthetic health records were populated using the official developer reference client, Health Connect Toolbox v2.3.3, via standard Jetpack SDK bindings to eliminate third-party implementation bias. While synthesized outcomes are reported in Table 1, full execution procedures, input payloads and evidence are compiled in the Empirical Verification Traceability Matrix in the Supplementary Section (42).

**Table 1:** Comparative analysis of technical features between Health Connect, Google Fit, and the Android Sensor API.

	Solutions		
Technical features	Health Connect	Google Fit	Android Sensor API
Storage and OS interaction	Considered in the Android security model. Verification of origin. *Evidence*: (9),${}^{13}$, TEST 0200 (OK), 0202 (OK)	Data source mechanism. *Evidence*: ${}^{12}$	Direct access to sensors. *Evidence*: (32)
Storage encryption	Encrypted storage. *Evidence*: ${}^{15,16}$, TEST 0207 (FAIL)	Cloud-based. *Evidence*: ${}^{17}$	(No storage/Real-time)
Operational model	Local operation. *Evidence*: ${}^{8}$, TEST 0262 (OK)	Cloud-based. *Evidence*: ${}^{17}$	Local operation. *Evidence*: ${}^{7}$
Data granularity and permissions	Granular permissions. *Evidence*: ${}^{8}$, TEST 0254 (OK), 0315 (OK)	General permissions. *Evidence*: ${}^{20}$	General permissions. *Evidence*: ${}^{19}$, (32)

##### 3.2.3.1 Storage and OS interaction

The interaction with the operating system varies significantly across the three solutions. In the case of the *Android Sensor API*, applications interact directly with hardware components through the SensorManager class (32), providing *Direct access to sensors* without an intermediate storage layer managed by the OS. In contrast, Google Fit operates as a platform where applications synchronise data, relying on external services rather than a direct OS integration. Nevertheless, it does implement some mechanisms to ensure traceability, such as the *Data source mechanism*, which allows the system to identify the app or the device that collects or transforms the data.^12^

Health Connect introduces a “container” architecture that centralises health information locally. Unlike the direct single-component interaction of the Sensor API and Google Fit, Health Connect requires a two-stage access model. This architecture of the data management process is explicitly *Considered in the Android security model* (9) and enables capabilities such as the *Verification of origin* to verify that only authorised applications can insert data.^13^ Empirical testing validated these structural claims. The execution of OWASP-MASTG-TEST-0200 and OWASP-MASTG-TEST-0202 (targeting MASVS-STORAGE-1 and MASVS-STORAGE-2) resulted in *PASSED*.^14^ These tests confirm that Health Connect completely avoids shared external storage and requests zero storage permissions, confining all records strictly to its private sandbox. While caller package validation is enforced at the IPC layer, this sandbox isolation provides essential indirect support for origin verification and data integrity by guaranteeing that third-party apps cannot bypass the Health Connect API gatekeeper through unmediated filesystem writes.

##### 3.2.3.2 Storage encryption

Security regarding how data is persisted is a key differentiator. The Android Sensor API does not provide a storage solution; it offers real-time access, leaving any subsequent storage security entirely up to the developer. Google Fit, being a cloud-based service, stores data outside the device. While this implies professional management by Google, it exposes information to potential vulnerabilities in the provider’s infrastructure (43), forcing users to trust how these risks are managed.

Finally, according to Android Developers documentation, “*The data in Health Connect is stored on-device and encrypted*”.^15^ However, the documentation does not clarify whether this encryption relies on Android’s native features or a custom implementation within Health Connect. Source code analysis confirms that data access is mediated via Inter-Process Communication (IPC), suggesting that encryption is likely managed at the OS level.^16^

To evaluate this, we executed OWASP-MASTG-TEST-0207 (evaluating local storage and runtime encryption under MASVS-STORAGE-1), which yielded a *FAIL*. We checked and noted that all inserted health records at the insternal database (healthcore.db) were in plaintext, so the test failed. For more information about the testing environment and execution of the tests please see the Empirical Verification Traceability Matrix in the Supplementary Section.

This highlights a critical architectural nuance. While the OS provides file-based encryption at rest, Health Connect lacks database-level encryption. Consequently, rooted devices or physical extractions fully expose health records in plaintext.

##### 3.2.3.3 Operational model

The operational mode defines the system’s reliance on external connectivity. The Android Sensor API and Health Connect both operate locally. The Sensor API has been available since early Android versions, allowing direct interaction with the device hardware. Health Connect maintains this local philosophy but acts as a unified repository to facilitate interoperability between applications without data leaving the device. Empirical validation via OWASP-MASTG-TEST-0262 (MASVS-STORAGE-2) confirmed a *PASSED* result, verifying that Health Connect’s backup configuration explicitly excludes all database files from cloud backups, permitting only direct device-to-device transfers. This restriction provides indirect architectural support for consent management and storage limitation by ensuring that sensitive health records do not silently replicate to remote cloud infrastructure, keeping data retention and user revocation strictly confined to the local device environment.

Conversely, Google Fit relies on a cloud-based model where data must be transmitted and stored externally. This outsourcing increases the attack surface (44) and limits the user’s direct control compared to local solutions. Notably, this cloud-based approach is being phased out, as Google Fit will be deprecated in 2026,^17^ while Health Connect will become mandatory for developers starting from 28th May 2025.^18^

##### 3.2.3.4 Data granularity and permissions

This feature represents the most substantial evolution in the Android ecosystem. In versions prior to Android 14, the Android Sensor API suffered from a lack of granularity: the BODY_SENSORS permission (32) granted access to all health sensors simultaneously (e.g., heart rate, blood pressure) even if the application only required one specific.^19^ In a similar way, Google Fit requires broad permissions to access health data categories.^20^ This “all-or-nothing” approach raised significant privacy and security concerns (45, 46).

Health Connect radically shifts this model by introducing fine-grained control. Access is no longer granted in bulk; instead, permissions are requested for specific pieces of data (e.g., READ_HEART_RATE). Furthermore, through the new Management Application (see Figure 3), users can manage read and write permissions independently for each app. For instance, a fitness app could be granted permission to write “calories consumed” but only read “calories burned,” significantly enhancing the level of control compared to the previous blanket permissions. The robustness of this mechanism was proven through OWASP-MASTG-TEST-0254 (MASVS-PRIVACY-1) and TEST-0315 (MASVS-PLATFORM-3), both of which returned *PASSED*.

**Figure 3:**
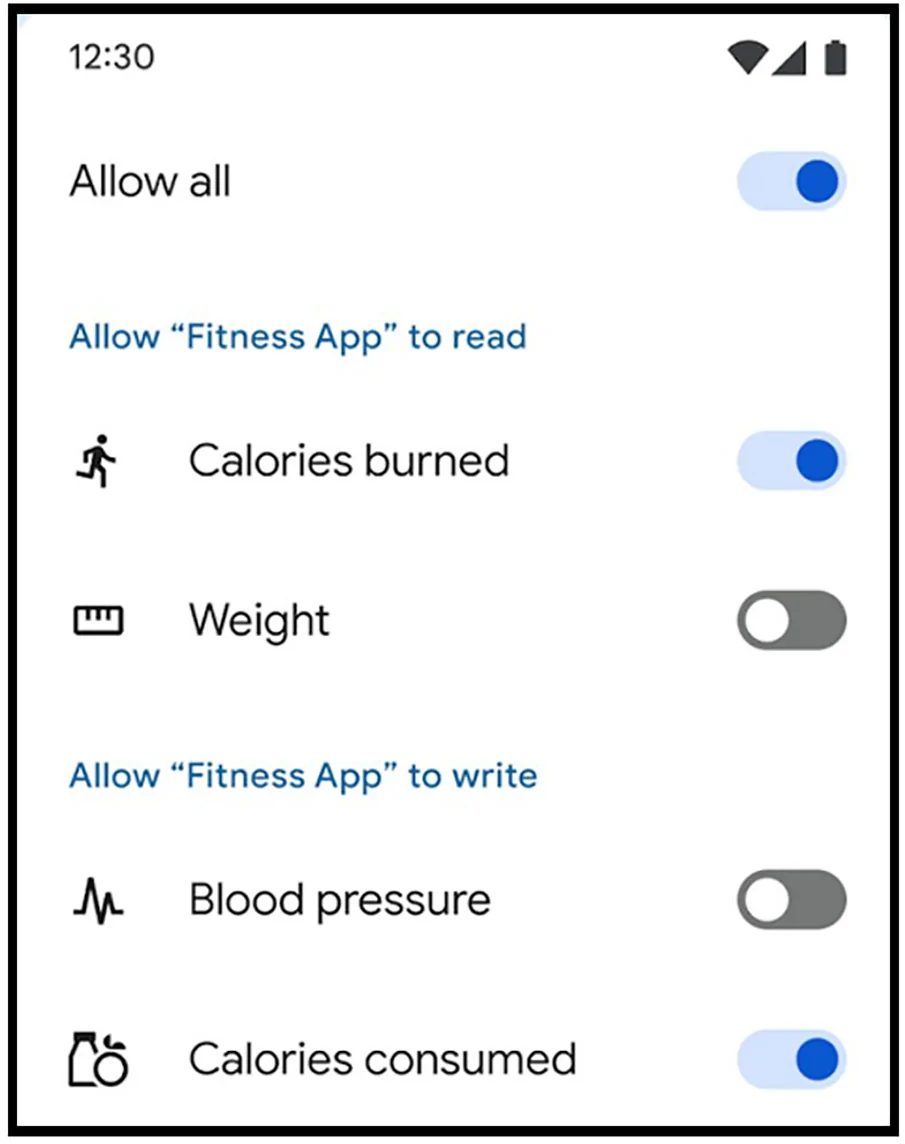
Data management within the Health Connect application.

Table 1 summarises the analysis of the technical features across the three health data management solutions in Android. Each solution exhibits distinct characteristics in terms of storage interaction, encryption, operational model, and data granularity, reflecting their evolution and differing approaches to health data management.

#### 3.2.4 Phase 4: API comparison

At this phase, Health Connect is compared with the previous Android health data management solutions identified in Section 3.2.2. This provides a comprehensive understanding of how it contributes to sensitive health data protection, and to determine whether it actually enhances protection.

To perform the comparison, we evaluate each API against the security and privacy criteria derived from the threat model established in Section 3.2.1. We assess how well the technical features of each API discussed in the previous phase (see Section 3.2.3) provide mechanisms to support each criterion.

To effectively summarise the comparative analysis, we present the results in (Tables 2, 3) that map the defined criteria against the different solutions evaluated. Under the premise established in our threat model (assuming a trusted local execution environment where the device and operating system remain uncompromised) we classify each API’s protection level using the four-tier evaluation scale defined in Section 3.2.1: *native support* (the API inherently provides and enforces the protection), *partial support* (the API provides safeguards with verifiable gaps or external dependencies), *developer-dependent* (the API delegates the entire security responsibility to the application developer), and *not supported* (the API fundamentally lacks the necessary mechanisms). Importantly, consistent with our role attribution of health APIs as data processors, these ratings evaluate technical and architectural support mechanisms provided at the API level to facilitate compliance, rather than denoting full or autonomous satisfaction of broad legal and operational requirements.

**Table 2:** Comparative security analysis between Health Connect, Google Fit, and the Android Sensor API.

	Solutions					
Security pillar	Health connect		Google fit		Android sensor API	
Confidentiality	**Partial support.**	Local operation. Granular permissions. Encrypted storage at rest. *Runtime gap: OWASP 0207.*	**Not supported.**	Cloud-based. General permissions.	**Developer-dependent.**	General permissions. (No native storage)
Integrity	**Native support.**	Local operation. Considered in the Android security model.	**Not supported.**	Cloud-based.	**Developer-dependent.**	Local operation. (Raw access requires dev safeguarding)
Availability	**Native support.**	Local operation.	**Not supported.**	Cloud-based.	**Native support.**	Local operation.
Authenticity	**Native support.**	Granular permissions. Verification of origin. Considered in the Android security model.	**Partial support.**	Data source mechanism. (External dependencies)	**Native support.**	Direct access to sensors.

Values in bold represent the result of the analysis for each pillar/criterion against the evaluated solution.

**Table 3:** Comparison of privacy between Health Connect, Google Fit, and Android Sensor API.

	Solutions					
Privacy criteria	Health Connect		Google Fit		Android Sensor API	
Data minimisation	**Native support.**	Granular permissions.	**Not supported.**	General permissions. Cloud-based.	**Not supported.**	General permission.
User’s Ability to Manage Consent	**Native support.**	Granular permissions. Local operation.	**Partial support.**	Device revoke available, but data persists in cloud.	**Developer-dependent.**	General permission. Local operation.
Transparency	**Native support.**	Granular permissions.	**Not supported.**	General permissions.	**Not supported.**	General permission.
Accuracy	**Native support.**	Verification of origin. Granular permissions.	**Partial support.**	Data source mechanism. Cloud sync latency.	**Native support.**	Direct access to sensors.
Storage limitation	**Native support.**	Management application.	**Partial support.**	Data source mechanism.	**Developer-dependent.**	Direct access to sensors. No native storage.

Values in bold represent the result of the analysis for each pillar/criterion against the evaluated solution.

##### 3.2.4.1 Security

The security analysis is founded upon a point-by-point comparison of each solution against the defined criteria (Confidentiality, Integrity, Availability, and Authenticity) built on top of the analysis performed at Section 3.2.3. The comparative results are presented in Table 2, detailing the evaluation of each criterion for Health Connect, Google Fit, and the Android Sensor API. The following points provide the reasoning for the assigned support level for each criterion:

- **Confidentiality.** Health Connect achieves *partial support*. While it offers local operation and granular access management (9), the empirical *FAIL* on runtime encryption (OWASP-MASTG-TEST-0207) indicates a residual risk within the app’s private directory. The cloud-based nature of Google Fit *does not support* confidentiality inherently, and the Android Sensor API is completely *developer-dependent*, leaving all protective implementation to the app creator (33, 34).
- **Integrity.** Health Connect achieves *native support* at the architectural level: by operating within an OS-isolated container and avoiding external storage, it natively protects stored records against unmediated file tampering by third-party apps (47), though semantic data validity downstream remains application-dependent. The Android Sensor API is *developer-dependent*: while local polling avoids transmission manipulation, it provides no native repository to safeguard acquired data. Google Fit is *not supported* as its cloud-based architecture exposes data in transit and remote storage to external vulnerabilities (43).
- **Availability.** Health Connect and the Android Sensor API offer *native support* from an architectural perspective by operating strictly on-device, insulating basic data access from internet outages or remote server downtimes (47), although device-level availability remains subject to local OS resource management. Google Fit is *not supported* natively, as operational availability is structurally dependent on network connectivity and third-party cloud infrastructure.
- **Authenticity.** Health Connect achieves *native support* by verifying authorized caller identities and recording source metadata through its OS-mediated container model (34). The Android Sensor API provides *native support* at the transport level by reading directly from local hardware drivers without intermediate proxies. Google Fit offers *partial support*: it provides an application-level data source mechanism^21^ for attribution, but remains reliant on cloud synchronization channels that introduce external spoofing risks.

Health Connect’s architectural shift towards local storage and granular permission control supports robust security. Although runtime encryption presents an area for improvement (rendering confidentiality as partial), it significantly addresses the inherent weaknesses of the cloud-based Google Fit and the lack of native guardrails in the Android Sensor API.

##### 3.2.4.2 Privacy

Similarly to what was outlined in Section 3.2.4.1, the privacy analysis is founded upon a point-by-point comparison of each solution against the previously defined criteria (Data Minimisation, User’s Ability to Manage Consent, Transparency, Accuracy, and Storage Limitation). The comparative results are presented in Table 3.

- **Data Minimisation.** Health Connect offers *native support* through its granular permission model, allowing access control at the individual data level. Both Google Fit and the Android Sensor API are *not supported* as they rely on single, general access permissions (such as BODY_SENSORS), preventing true minimisation.
- **Consent.** Health Connect provides *native support* through granular, local control mechanisms via the Management App. The Android Sensor API is *developer-dependent*, as the OS provides the toggle, but respecting the revocation downstream relies on the app. Google Fit achieves *partial support*: while local permissions can be revoked, it provides no guarantee that data already stored remotely will stop being processed.
- **Transparency.** Evaluated from a technical processor standpoint, Health Connect provides *native support* through its granular permission framework, offering users technical transparency over the precise health data categories requested (e.g., heart rate). However, this represents architectural support rather than full GDPR transparency compliance, which requires controllers to provide comprehensive privacy notices and legal disclosures. Google Fit and the Android Sensor API are *not supported* as their coarse-grained permissions obscure the specific scope of collected data.
- **Accuracy.** Health Connect offers *native support* through technical origin attribution and API endpoints that allow authorized updates and rectifications to stored records. The Android Sensor API provides *native support* for immediate operational accuracy by delivering raw, real-time telemetry directly from hardware without transmission latency, though calibration accuracy remains sensor-dependent. Google Fit provides *partial support*; while offering data attribution tools, cloud synchronization latency and offline caching inconsistencies can compromise the real-time exactness of health metrics.
- **Storage Limitation.** Health Connect provides *native support* at the API and OS level by supplying built-in retention mechanisms: users can configure data deletion via the management application, while backup rules prevent uncontrolled remote persistence. Google Fit achieves *partial support* by offering cloud history management tools,^22^ though data lifespan remains coupled to remote cloud accounts. Conversely, the Android Sensor API is *developer-dependent*: by providing raw access without an intermediary layer, the responsibility of deleting or limiting data falls entirely on the developer.

Health Connect represents the most significant privacy advancement in the ecosystem. Its native support across all the privacy domains analysed directly addresses the core failings of its predecessors.

## 4 Discussion

The analysis conducted has shown that Health Connect represents an improvement compared to previous solutions used by health applications, both in terms of security and privacy, which answers the research question posed at the outset. The selection of these specific security and privacy criteria in Section 3.2.1—grounded in the GDPR, NIS2 Directive, and CRA—provides a suitable framework to characterise the baseline robustness of Android Health APIs because they capture the foundational mechanisms data processors must provide. These criteria cover the core architectural controls (e.g., data minimisation, encryption and granular access) needed for regulatory compliance and mitigating common mobile health risks.

However, the question arises as to what extent these improvements are a result of the Health Connect solution itself, or whether they stem from a better utilisation of the capabilities already offered by the Android operating system. In our view, the specific improvements that can be exclusively attributed to Health Connect include the increased granularity of health data permissions and the two-phase access control, which is facilitated by the design of this “container” architecture. The increase in granularity is a significant advancement in terms of privacy, as it facilitates the minimisation of shared data. However, this aspect does not directly impact data security.

On the other hand, security improvements stem from decisions such as the design of Health Connect and encryption. As mentioned in Section 3.2.3, this design deviates from the previously followed Android model, which involved a single access layer where each permission granted direct access to a resource. Now, this model is reinforced with two levels of protection, suggesting that Android developers have opted to revise their approach to strengthen security, a change likely to be extended to other types of data in the future. Another crucial factor in the security improvements is encryption. As noted in Section 3.2.3, it seems that this represents a better utilisation of the operating system’s functionalities rather than an innovative contribution from Health Connect itself. This highlights to developers the importance not only of producing high-quality independent work but also of maximising the latest features provided by operating systems, which enables them to achieve better solutions more easily.

The scope of our analysis is centered on the intrinsic architectural capabilities of the APIs themselves, rather than conducting a full security and privacy audit or evaluating the behavior of real-world end-user applications. A comprehensive study of the guarantees offered by a specific application would require examining all ecosystem elements, including third-party communications and data collection mechanisms. Also, while empirical validation (using OWASP MASTG) was conducted for Health Connect, the cloud-based security of Google Fit was assessed based on references and documentation rather than through experimental penetration testing. Furthermore, as indicated in Section 3.2.4.2, the selected privacy criteria were filtered to include only those that are technically verifiable, though the remaining criteria defined in official guidelines (5, 16), such as the *purpose limitation*, are also relevant when evaluating overall privacy.

The present study synthesizes insights derived from publicly available official documentation, which, while comprehensive in outlining architectural principles, offers limited granularity regarding specific implementation mechanisms. The absence of detailed technical specifications, particularly concerning the cryptographic protocols employed by Health Connect to ensure privacy and security, represents a significant area where further scholarly and technical disclosure would enhance the evaluative framework. Addressing such informational gaps is crucial for fostering transparency, thereby facilitating informed decision-making and promoting widespread adoption of this emerging API. Furthermore, Google has a history of delays in meeting deadlines and implementing scheduled changes, as happened with the adoption of the data security section in Google Play.^23^ Something similar could occur with the announcement that the Google Fit API will no longer be used, potentially hindering Health Connect’s adoption.

Regarding external validity, the proposed methodology was intentionally designed to be technology-agnostic. Because the assessment framework is built upon universal threat modeling principles and broad regulatory requirements rather than OS-specific code implementations, it can be readily adapted to evaluate health data management systems in other mobile ecosystems, such as Apple’s HealthKit, or applied to future iterations of Android health APIs. For instance, when applying this methodology to Apple HealthKit, health data can be mapped to its specific *Characteristic data* and *Sample data* types, authenticity and source mechanisms can be evaluated through its *Source data* tracking, and storage limitation can be studied via its handling of *Deleted objects*.^24^ Furthermore, the empirical validation strategy can be seamlessly ported to iOS, as the OWASP Mobile Application Security Testing Guide (MASTG) provides platform-specific tests natively designed for Apple environments (e.g., iOS Sandbox and Keychain verification), allowing the same security standards used for Android to be rigorously applied to evaluate Apple’s architecture.

## 5 Conclusions

This study makes two primary contributions to the field of mobile health security and privacy. First, it proposes a general, technology-agnostic methodology that can be applied to evaluate the mechanisms used for managing users’ health data across any mobile operating system. Second, it practically applies this methodology to assess the security and privacy of Health Connect, providing a comprehensive comparison with prior health data management solutions in the Android ecosystem, namely Google Fit and the Android Sensors API.

In response to the initial research question, the analysis—supported by partial empirical validation using established mobile application security testing standards—shows that Health Connect substantially advances protection by providing mechanisms to help developers improve security and privacy. In terms of security, it provides native support mechanisms for integrity, availability and authenticity, while offering partial support for confidentiality, as it fails in runtime encryption. Regarding privacy, it provides native support mechanisms across all evaluated criteria (data minimisation, user consent management, transparency, accuracy, and storage limitation), directly overcoming the developer-dependent and unsupported mechanisms characteristic of legacy APIs. Table 4 synthesises these findings, illustrating the degree of support—native, partial, developer-dependent or not supported—each solution provides across the defined criteria.

**Table 4:** Consolidated security and privacy analysis: Health Connect, Google Fit, and Android Sensor API.

	Solutions		
Criteria	Health Connect	Google Fit	Android Sensor API
Security criteria			
Confidentiality	Partial support	Not supported	Developer-dependent
Integrity	Native support	Not supported	Developer-dependent
Availability	Native support	Not supported	Native support
Authenticity	Native support	Partial support	Native support
Privacy criteria			
Data Minimisation	Native support	Not supported	Not supported
User’s Ability to Manage Consent	Native support	Partial support	Developer-dependent
Transparency	Native support	Not supported	Not supported
Accuracy	Native support	Partial support	Native support
Storage Limitation	Native support	Partial support	Developer-dependent

Despite these advancements, empirical evaluation revealed notable gaps, such as the failure to enforce runtime encryption within the application’s private directory, leaving residual risks in unencrypted local memory. This underscores the need for greater clarity in Health Connect’s official documentation, particularly regarding the exact mechanisms and scope of its encryption implementation, which is essential to foster developer trust and promote adoption. In summary, while Health Connect marks an important step forward in the secure management of health data on Android, addressing technical limitations like runtime encryption and improving documentation transparency remain vital.

### 5.1 Future work

Since the current study successfully applied OWASP MASVS controls (such as MASVS-STORAGE, MASVS-CRYPTO-2, and MASVS-PRIVACY) to evaluate Health Connect’s architectural baseline, future work should extend this empirical validation to real-world, end-user mHealth applications. Specifically, investigating how developers implement compensating controls for the runtime encryption vulnerabilities identified during this analysis would provide deeper insights into the practical security posture of the Android health ecosystem. Furthermore, applying this technology-agnostic methodology to evaluate equivalent APIs in other platforms, such as Apple’s HealthKit, represents a logical next step to establish comprehensive cross-platform security and privacy benchmarks.

Another key objective for future work involves expanding the set of privacy criteria utilised in this analysis. By incorporating a more diverse range of metrics, such as the procedural and governance requirements established by supervisory authority guidelines (5, 16), future studies can provide a more holistic assessment of data protection risks, user consent workflows, and overall regulatory compliance.

## Data Availability

The datasets presented in this study can be found in online repositories. The names of the repository/repositories and accession number(s) can be found in the article/Supplementary Material.

## Footnotes

^1^ https://hackernoon.com/google-io-2022-key-announcements-for-developers

^2^See the “App info” section at https://play.google.com/store/apps/details?id=com.google.android.apps.healthdata

^3^ https://developer.android.com/health-and-fitness/health-connect/migration/android-13-to-14

^4^ https://support.google.com/googleplay/android-developer/answer/16558241

^5^ https://developer.android.com/guide/topics/permissions/overview

^6^All analysed files are available at Supplementary Section.

^7^ https://android.googlesource.com/platform/frameworks/base/+/refs/heads/master/core/java/android/hardware/SensorManager.java

^8^ https://android.googlesource.com/platform//frameworks/support/+/refs/heads/androidx-health-connect-release/health/connect/connect-client/

^9^ https://developer.android.com/s/results?q=health+management

^10^ https://developer.android.com/health-and-fitness/health-connect/

^11^ https://developer.android.com/health-and-fitness/health-connect/migration/fit

^12^ https://developers.google.com/fit/android/data-attribution?hl=en

^13^ https://developer.android.com/health-and-fitness/health-connect/metadata?hl=en

^14^In OWASP testing, *PASSED* or *OK* indicates the security tests were successfully passed; *FAIL* means they were not.

^15^ https://developer.android.com/codelabs/health-connect#0

^16^ https://android.googlesource.com/platform//frameworks/support/+/refs/heads/androidx-health-connect-release/health/connect/connect-client/src/main/java/androidx/health/connect/client/HealthConnectClient.kt

^17^ https://developers.google.com/fit/android

^18^ https://support.google.com/googleplay/android-developer/table/12921780?hl=en

^19^ https://developer.android.com/reference/android/Manifest.permission#BODY_SENSORS

^20^ https://developers.google.com/fit/android/authorization

^21^ https://developers.google.com/fit/android/data-attribution?hl=en

^22^ https://support.google.com/fit/answer/9592657

^23^ https://support.google.com/googleplay/android-developer/answer/10787469

^24^ https://developer.apple.com/documentation/healthkit/about-the-healthkit-framework
