# Supplementary material for: On the evolution of security and privacy in Android health APIs
Source: Front Digit Health. 2026 Oct 28;8:1818536. doi: 10.3389/fdgth.2026.1818536 (PMC13751995; doi:10.3389/fdgth.2026.1818536)
Supplement: Supplementary file 1 [file Datasheet1.zip › supplemental-data/documentation-googlefit-data-trazability-1.pdf]

The Google Fit APIs, including the Google Fit REST API, **will be deprecated in 2026**. As of May 1, 2024, **developers cannot sign up to use these APIs**.

For instructions on which API or platform to migrate to, visit the Health Connect migration guide (https://developer.android.com/guide/health-and-fitness/health-connect-guidelines/migrate/migration-guide). For a comparison of Health Connect with the Google Fit APIs and the Fitbit Web APIs, visit the Health Connect comparison guide (https://developer.android.com/guide/health-and-fitness/health-connect-guidelines/migrate/comparison-guide).

Learn more about Health Connect (https://developer.android.com/guide/health-and-fitness/health-connect) and how to integrate with the API.

Data attribution

AI-generated Key Takeaways

Every data point in Google Fit has an associated data source. Data sources contain information that identifies the app or the device that collects or transforms the data. The package name of the app is available for data sources that don't represent a physical sensor.

Google Fit lets you do the following:

- Invoke an intent to view data that's associated with a specific app.
- Receive intents to show data using your app.
- Find out which app inserted a session (/fit/sessions). For more information, see Work with sessions (/fit/android/using-sessions).

Determine which app inserted a data point

To obtain the package name of the application that inserted a data point, first call **`DataPoint.getOriginalDataSource`** (/android/reference/com/google/android/gms/fitness/data/DataPoint#public-datasource-getoriginaldatasource) to get the data source, then call the **`DataSource.getAppPackageName`** (/android/reference/com/google/android/gms/fitness/data/DataSource#public-string-getapppackagename) method:

Kotlin (#kotlin) Java (#java)

```
val dataPoint : DataPoint = ...
val dataSource = dataPoint.originalDataSource
val appPkgName = dataSource.appPackageName
```

Receive intents from other apps

To register your app to receive intents from other health and wellness apps, declare an intent filter in your manifest that's similar to the following:

```
<intent-filter>
    <action android:name="vnd.google.fitness.VIEW" />
    <data android:mimeType="vnd.google.fitness.data_type/com.google.step_count.cumulative" />
    <data android:mimeType="vnd.google.fitness.data_type/com.google.step_count.delta" />
</intent-filter>
```

Note: Don't include the **`DEFAULT`** category in your intent filter, unless you want your app to receive intents for data created by other apps.

Each intent that your app receives from Google Fit is of only one type, but you can filter for multiple MIME types in a single intent filter. Your app's intent filter needs to include all of the data types that your app supports, including custom data types.

The fitness intents include the following extras:

- `vnd.google.gms.fitness.start_time`
- `vnd.google.gms.fitness.end_time`
- `vnd.google.gms.fitness.data_source`

You can obtain data from these extras as follows:

Kotlin (#kotlin) Java (#java)

```
override fun onCreate(savedInstanceState: Bundle?) {
    super.onCreate(savedInstanceState)
    ...
    val supportedType = DataType.getMimeType(DataType.TYPE_STEP_COUNT_DELTA)

    if (Intent.ACTION_VIEW == intent.action && supportedType == intent.type) {
        // Get the intent extras
        val startTime = Fitness.getStartTime(intent, TimeUnit.MILLISECONDS);
        val endTime = Fitness.getEndTime(intent, TimeUnit.MILLISECONDS)
        val dataSource = DataSource.extract(intent)
    }
}
```

To obtain the MIME type for a custom data type, use the `MIME_TYPE_PREFIX` (/android/reference/com/google/android/gms/fitness/data/DataType#public-static-final-string-mime_type_prefix) constant:

Kotlin (#kotlin) Java (#java)

```
val supportedType = DataType.MIME_TYPE_PREFIX + "com.company.customdatatype"
```

Invoke an intent to view data

To invoke an intent to view data with another app, use the `HistoryApi.ViewIntentBuilder` (/android/reference/com/google/android/gms/fitness/HistoryApi.ViewIntentBuilder) class:

Kotlin (#kotlin) Java (#java)

```
// Inside your activity
val startTime = ...
val endTime = ...
val dataSource = ...
val dataType = ...

val fitIntent = HistoryApi.ViewIntentBuilder(this, dataType)
    .setTimeInterval(startTime, endTime, TimeUnit.MILLISECONDS)
    .setDataSource(dataSource) // Optional if a specific data source is desired
    .setPreferredApplication("com.example.app") // Optional if you'd like a
    // specific app to handle the intent if that app is installed on the device
    .build()
```

Learn more about how to use intents and intent filters (https://developer.android.com/guide/components/intents-filters).

Except as otherwise noted, the content of this page is licensed under the Creative Commons Attribution 4.0 License (https://creativecommons.org/licenses/by/4.0/), and code samples are licensed under the Apache 2.0 License (https://www.apache.org/licenses/LICENSE-2.0). For details, see the Google Developers Site Policies (https://developers.google.com/site-policies). Java is a registered trademark of Oracle and/or its affiliates.

Last updated 2025-08-28 UTC.
