# Supplementary material for: On the evolution of security and privacy in Android health APIs
Source: Front Digit Health. 2026 Oct 28;8:1818536. doi: 10.3389/fdgth.2026.1818536 (PMC13751995; doi:10.3389/fdgth.2026.1818536)
Supplement: Supplementary file 1 [file Datasheet1.zip › supplemental-data/documentation-googlefit-general-1.pdf]

The Google Fit APIs, including the Google Fit REST API, **will be deprecated in 2026**. As of May 1, 2024, **developers cannot sign up to use these APIs**.

For instructions on which API or platform to migrate to, visit the Health Connect migration guide (https://developer.android.com/guide/health-and-fitness/health-connect-guidelines/migrate/migration-guide). For a comparison of Health Connect with the Google Fit APIs and the Fitbit Web APIs, visit the Health Connect comparison guide (https://developer.android.com/guide/health-and-fitness/health-connect-guidelines/migrate/comparison-guide).

Learn more about Health Connect (https://developer.android.com/guide/health-and-fitness/health-connect) and how to integrate with the API.

Android APIs

AI-generated Key Takeaways

The Google Fit APIs for Android are part of Google Play services (/android/guides/overview). The Google Fit APIs are supported on Android 4.1 (API level 16) and higher. Using these APIs, your app can do the following:

- Read near-real-time and historic data, including data from Bluetooth Low Energy (BLE) devices.
- Record activities.
- Associate data with a session.
- Set fitness goals.

Read sensor data

If your app provides information about a user's activity, such as their daily steps, it can be helpful to show near-real-time data about the user's movements. The Sensors API (/fit/android/sensors) provides access to streams of raw data that contain this information. The data can come from sensors available on the Android-powered device as well as from sensors in companion devices, such as wearables.

Record data

If your app needs to gather, store, and sync data about a user's physical activity, you can use the Recording API (/fit/android/record). This API stores health and wellness data using subscriptions. Your app specifies one or more types of activities to record. Google Fit then stores data of the specified types in the background even when your app isn't running. It also restores these subscriptions when the system is restarted.

Historical data

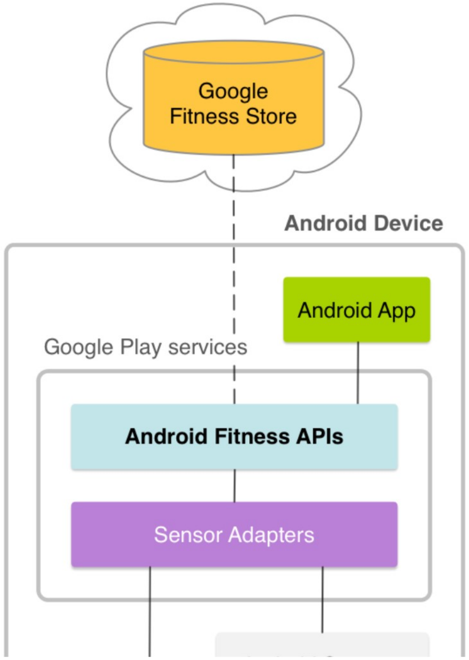

If you want users to be able to view fitness data from past activities, you can use the History API (/fit/android/history). This API provides access to the historical health and wellness data and lets apps perform bulk operations, such as inserting, deleting, and reading data.

Sessions

Fitness data can be beneficial by itself, but the data is more useful when you combine it with additional information about the user's activity. The Sessions API (/fit/android/using-sessions) bundles health and wellness data, along with some metadata, into units called *sessions*. Sessions represent a time interval during which the user performs a fitness activity.

View and set goals

Your app can help motivate users by showing how their fitness activity compares to the goals that they've set. The Goals API (/fit/android/goals) helps your app show users the health and fitness goals that they've created.

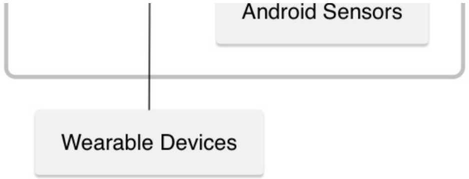

Figure 1. *Google Fit on Android.*

Except as otherwise noted, the content of this page is licensed under the Creative Commons Attribution 4.0 License (https://creativecommons.org/licenses/by/4.0/), and code samples are licensed under the Apache 2.0 License (https://www.apache.org/licenses/LICENSE-2.0). For details, see the Google Developers Site Policies (https://developers.google.com/site-policies). Java is a registered trademark of Oracle and/or its affiliates.

Last updated 2025-08-28 UTC.
