# Supplementary material for: On the evolution of security and privacy in Android health APIs
Source: Front Digit Health. 2026 Oct 28;8:1818536. doi: 10.3389/fdgth.2026.1818536 (PMC13751995; doi:10.3389/fdgth.2026.1818536)
Supplement: Supplementary file 1 [file Datasheet1.zip › supplemental-data/documentation-googlefit-permissions.pdf]

The Google Fit APIs, including the Google Fit REST API, **will be deprecated in 2026**. As of May 1, 2024, **developers cannot sign up to use these APIs**.

For instructions on which API or platform to migrate to, visit the Health Connect migration guide (https://developer.android.com/guide/health-and-fitness/health-connect-guidelines/migrate/migration-guide). For a comparison of Health Connect with the Google Fit APIs and the Fitbit Web APIs, visit the Health Connect comparison guide (https://developer.android.com/guide/health-and-fitness/health-connect-guidelines/migrate/comparison-guide).

Learn more about Health Connect (https://developer.android.com/guide/health-and-fitness/health-connect) and how to integrate with the API.

Android Permissions

AI-generated Key Takeaways

Android permissions (https://developer.android.com/guide/topics/permissions/overview) aim to protect the privacy of an Android user. They prompt the user when apps want to access data types for sensitive data like contacts or photos, and system features like location or step detection. Users grant these permissions when first downloading your app.

If your app needs access to any of these data types (#data_types_that_need_android_permissions) (a subset of the Google Fit data types), request the relevant Android permission before requesting the OAuth permissions. See below (#requesting_android_permissions).

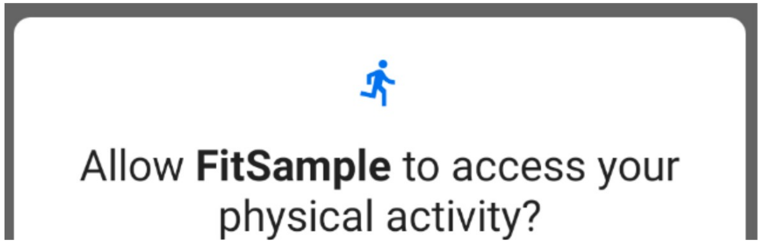

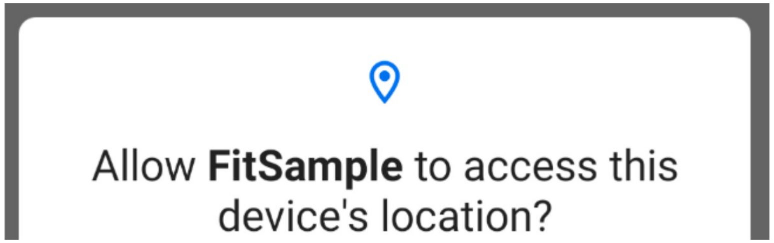

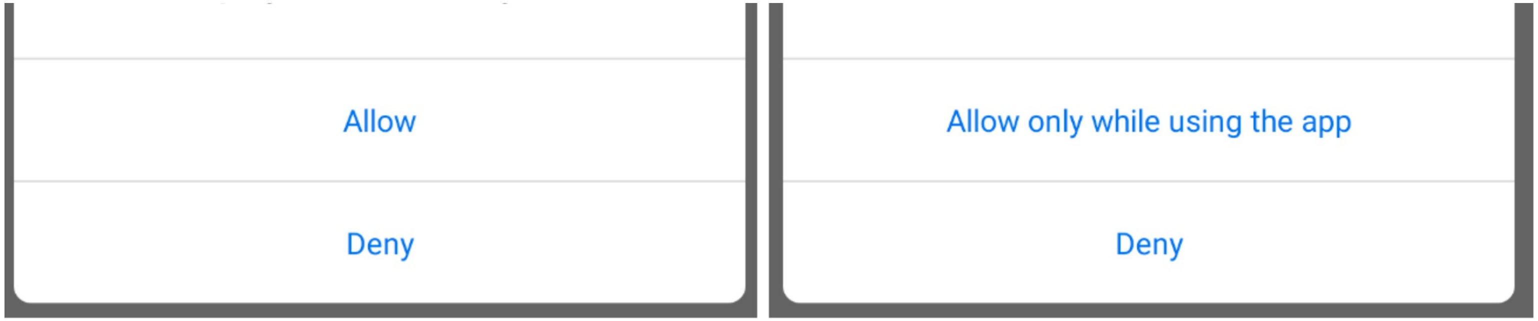

Figure 1. *The Android permissions consent screen.*

Data types that need Android permissions

To access these physical activity data types, you'll need to request the `ACTIVITY_RECOGNITION` Android permission (#requesting_android_permissions):

- To record (/android/reference/com/google/android/gms/fitness/RecordingClient) these data types:
  - `com.google.step_count.delta`
  - `com.google.step_count.cumulative`
  - `com.google.step_count.cadence`
  - `com.google.activity.segment`
  - `com.google.calories.expended`
- To read (/android/reference/com/google/android/gms/fitness/HistoryClient) these data types:
  - `com.google.step_count.delta`

- `com.google.step_count.cumulative`
- `com.google.step_count.cadence`
- `com.google.activity.segment`
- `com.google.activity.exercise`

To access these data types, you'll need to request the `ACCESS_FINE_LOCATION` Android permission (https://developer.android.com/reference/android/Manifest.permission.html#ACCESS_FINE_LOCATION):

- To read these data types:
  - `com.google.distance.delta`
  - `com.google.location.sample`
  - `com.google.location.bounding_box`
  - `com.google.speed`

To record (/android/reference/com/google/android/gms/fitness/RecordingClient) these data types, you'll need to request the `BODY_SENSORS` Android permission (https://developer.android.com/reference/android/Manifest.permission.html#BODY_SENSORS):

- `com.google.heart_rate.bpm`

Requesting Android permissions

Learn about requesting Android permissions (https://developer.android.com/training/permissions/requesting), the physical activity recognition permission (https://developer.android.com/about/versions/10/privacy/changes#physical-activity-recognition), the fine location permission (https://developers.google.com/maps/documentation/android-sdk/location#location_permissions), and the body sensors permission

(https://developer.android.com/reference/android/Manifest.permission.html#BODY_SENSORS).

To access the data types above with the Google Fit APIs, you'll need to implement logic to handle requesting Android permissions for both Android 10 and previous versions of Android. These examples use the `ACTIVITY_RECOGNITION` (https://developer.android.com/reference/android/Manifest.permission#ACTIVITY_RECOGNITION) permission.

Android 10Android 9 and below...
(#android-10)

So your app can target API level 29 or above, request the permission from the user, and register the permission in the application manifest file.

1. Add the permission to the manifest file (https://developer.android.com/training/permissions/requesting#perm-add).

```
<uses-permission android:name="android.permission.ACTIVITY_RECOGNITION"/>
```

2. Check if the permission is granted (https://developer.android.com/training/permissions/requesting#perm-check):

```
if (ContextCompat.checkSelfPermission(thisActivity, Manifest.permission.ACTIVITY_RECOGNITION)
          != PackageManager.PERMISSION_GRANTED) {
      // Permission is not granted
}
```

3. If permission isn't already granted, request the permission:

```
ActivityCompat.requestPermissions(thisActivity,
```

```
            arrayOf(Manifest.permission.ACTIVITY_RECOGNITION),
            MY_PERMISSIONS_REQUEST_ACTIVITY_RECOGNITION)
```

Except as otherwise noted, the content of this page is licensed under the Creative Commons Attribution 4.0 License (https://creativecommons.org/licenses/by/4.0/), and code samples are licensed under the Apache 2.0 License (https://www.apache.org/licenses/LICENSE-2.0). For details, see the Google Developers Site Policies (https://developers.google.com/site-policies). Java is a registered trademark of Oracle and/or its affiliates.

Last updated 2025-08-28 UTC.
