# Supplementary material for: On the evolution of security and privacy in Android health APIs
Source: Front Digit Health. 2026 Oct 28;8:1818536. doi: 10.3389/fdgth.2026.1818536 (PMC13751995; doi:10.3389/fdgth.2026.1818536)
Supplement: Supplementary file 1 [file Datasheet1.zip › supplemental-data/documentation-health-connect-permissions.pdf]

Health Permissions

Added in API level 34 (/guide/topics/manifest/uses-sdk-element#ApiLevels)

Kotlin (/reference/kotlin/android/health/connect/HealthPermissions) | **Java**

```
public final class HealthPermissions
extends Object
```
(/reference/java/lang/Object)

java.lang.Object (/reference/java/lang/Object)
↳ android.health.connect.HealthPermissions

Permissions for accessing the HealthConnect APIs.

Apps must support `Intent.ACTION_VIEW_PERMISSION_USAGE` (/reference/android/content/Intent#ACTION_VIEW_PERMISSION_USAGE) with `HealthConnectManager.CATEGORY_HEALTH_PERMISSIONS` (/reference/android/health/connect/HealthConnectManager#CATEGORY_HEALTH_PERMISSIONS) category to be granted read/write health data permissions.

Summary

Constants

`String` (/reference/java/lang/String)	`READ_ACTIVE_CALORIES_BURNED` (/reference/android/health/connect/HealthPermissions#READ_ACTIVE_CALORIES_BURNED)<br>Allows an application to read the user's active calories burned data.

`String` (/reference/java/lang/String)	`READ_ACTIVITY_INTENSITY` (/reference/android/health/connect/HealthPermissions#READ_ACTIVITY_INTENSITY)<br><br>Allows an application to read the user's activity intensity data.
`String` (/reference/java/lang/String)	`READ_BASAL_BODY_TEMPERATURE` (/reference/android/health/connect/HealthPermissions#READ_BASAL_BODY_TEMPERATURE)<br><br>Allows an application to read the user's body temperature data.
`String` (/reference/java/lang/String)	`READ_BASAL_METABOLIC_RATE` (/reference/android/health/connect/HealthPermissions#READ_BASAL_METABOLIC_RATE)<br><br>Allows an application to read the user's basal metabolic rate data.
`String` (/reference/java/lang/String)	`READ_BLOOD_GLUCOSE` (/reference/android/health/connect/HealthPermissions#READ_BLOOD_GLUCOSE)<br><br>Allows an application to read the user's blood glucose data.
`String` (/reference/java/lang/String)	`READ_BLOOD_PRESSURE` (/reference/android/health/connect/HealthPermissions#READ_BLOOD_PRESSURE)<br><br>Allows an application to read the user's blood pressure data.
`String` (/reference/java/lang/String)	`READ_BODY_FAT` (/reference/android/health/connect/HealthPermissions#READ_BODY_FAT)<br><br>Allows an application to read the user's body fat data.
`String` (/reference/java/lang/String)	`READ_BODY_TEMPERATURE` (/reference/android/health/connect/HealthPermissions#READ_BODY_TEMPERATURE)<br><br>Allows an application to read the user's body temperature data.

`String` (/reference/java/lang/String)	`READ_BODY_WATER_MASS` (/reference/android/health/connect/HealthPermissions#READ_BODY_WATER_MASS)<br><br>Allows an application to read the user's body water mass data.
`String` (/reference/java/lang/String)	`READ_BONE_MASS` (/reference/android/health/connect/HealthPermissions#READ_BONE_MASS)<br><br>Allows an application to read the user's bone mass data.
`String` (/reference/java/lang/String)	`READ_CERVICAL_MUCUS` (/reference/android/health/connect/HealthPermissions#READ_CERVICAL_MUCUS)<br><br>Allows an application to read the user's cervical mucus data.
`String` (/reference/java/lang/String)	`READ_DISTANCE` (/reference/android/health/connect/HealthPermissions#READ_DISTANCE)<br><br>Allows an application to read the user's distance data.
`String` (/reference/java/lang/String)	`READ_ELEVATION_GAINED` (/reference/android/health/connect/HealthPermissions#READ_ELEVATION_GAINED)<br><br>Allows an application to read the user's elevation gained data.
`String` (/reference/java/lang/String)	`READ_EXERCISE` (/reference/android/health/connect/HealthPermissions#READ_EXERCISE)<br><br>Allows an application to read the user's exercise data.

**String** (/reference/java/lang/String)	**READ_EXERCISE_ROUTES** (/reference/android/health/connect/HealthPermissions#READ_EXERCISE_ROUTES)<br><br>Allows an application to read **ExerciseRoute** (/reference/android/health/connect/datatypes/ExerciseRoute).
**String** (/reference/java/lang/String)	**READ_FLOORS_CLIMBED** (/reference/android/health/connect/HealthPermissions#READ_FLOORS_CLIMBED)<br><br>Allows an application to read the user's floors climbed data.
**String** (/reference/java/lang/String)	**READ_HEALTH_DATA_HISTORY** (/reference/android/health/connect/HealthPermissions#READ_HEALTH_DATA_HISTORY)<br><br>Allows an application to read the entire history of health data (of any type).
**String** (/reference/java/lang/String)	**READ_HEALTH_DATA_IN_BACKGROUND** (/reference/android/health/connect/HealthPermissions#READ_HEALTH_DATA_IN_BACKGROUND)<br><br>Allows an application to read health data (of any type) in background.
**String** (/reference/java/lang/String)	**READ_HEART_RATE** (/reference/android/health/connect/HealthPermissions#READ_HEART_RATE)<br><br>Allows an application to read the user's heart rate data.
**String** (/reference/java/lang/String)	**READ_HEART_RATE_VARIABILITY** (/reference/android/health/connect/HealthPermissions#READ_HEART_RATE_VARIABILITY)<br><br>Allows an application to read the user's heart rate variability data.

`String` (/reference/java/lang/String)	`READ_HEIGHT` (/reference/android/health/connect/HealthPermissions#READ_HEIGHT)<br>Allows an application to read the user's height data.
`String` (/reference/java/lang/String)	`READ_HYDRATION` (/reference/android/health/connect/HealthPermissions#READ_HYDRATION)<br>Allows an application to read the user's hydration data.
`String` (/reference/java/lang/String)	`READ_INTERMENSTRUAL_BLEEDING` (/reference/android/health/connect/HealthPermissions#READ_INTERMENSTRUAL_BLEEDING)<br>Allows an application to read the user's intermenstrual bleeding data.
`String` (/reference/java/lang/String)	`READ_LEAN_BODY_MASS` (/reference/android/health/connect/HealthPermissions#READ_LEAN_BODY_MASS)<br>Allows an application to read the user's lean body mass data.
`String` (/reference/java/lang/String)	`READ_MEDICAL_DATA_ALLERGIES_INTOLERANCES` (/reference/android/health/connect/HealthPermissions#READ_MEDICAL_DATA_ALLERGIES_INTOLERANCES)<br>Allows an application to read the user's data about allergies and intolerances.
`String` (/reference/java/lang/String)	`READ_MEDICAL_DATA_CONDITIONS` (/reference/android/health/connect/HealthPermissions#READ_MEDICAL_DATA_CONDITIONS)<br>Allows an application to read the user's data about medical conditions.
`String` (/reference/java/lang/String)	`READ_MEDICAL_DATA_LABORATORY_RESULTS` (/reference/android/health/connect/HealthPermissions#READ_MEDICAL_DATA_LABORATORY_RESULTS)<br>Allows an application to read the user's laboratory result data.

`String` (/reference/java/lang/String)	`READ_MEDICAL_DATA_MEDICATIONS` (/reference/android/health/connect/HealthPermissions#READ_MEDICAL_DATA_MEDICATIONS)<br><br>Allows an application to read the user's medication data.
`String` (/reference/java/lang/String)	`READ_MEDICAL_DATA_PERSONAL_DETAILS` (/reference/android/health/connect/HealthPermissions#READ_MEDICAL_DATA_PERSONAL_DETAILS)<br><br>Allows an application to read the user's personal details.
`String` (/reference/java/lang/String)	`READ_MEDICAL_DATA_PRACTITIONER_DETAILS` (/reference/android/health/connect/HealthPermissions#READ_MEDICAL_DATA_PRACTITIONER_DETAILS)<br><br>Allows an application to read the user's data about the practitioners who have interacted with them in their medical record.
`String` (/reference/java/lang/String)	`READ_MEDICAL_DATA_PREGNANCY` (/reference/android/health/connect/HealthPermissions#READ_MEDICAL_DATA_PREGNANCY)<br><br>Allows an application to read the user's pregnancy data.
`String` (/reference/java/lang/String)	`READ_MEDICAL_DATA_PROCEDURES` (/reference/android/health/connect/HealthPermissions#READ_MEDICAL_DATA_PROCEDURES)<br><br>Allows an application to read the user's data about medical procedures.
`String` (/reference/java/lang/String)	`READ_MEDICAL_DATA_SOCIAL_HISTORY` (/reference/android/health/connect/HealthPermissions#READ_MEDICAL_DATA_SOCIAL_HISTORY)<br><br>Allows an application to read the user's social history data.
`String` (/reference/java/lang/String)	`READ_MEDICAL_DATA_VACCINES` (/reference/android/health/connect/HealthPermissions#READ_MEDICAL_DATA_VACCINES)<br><br>Allows an application to read the user's data about immunizations and

	vaccinations.
**String** (/reference/java/lang/String)	**READ_MEDICAL_DATA_VISITS** (/reference/android/health/connect/HealthPermissions#READ_MEDICAL_DATA_VISITS)<br><br>Allows an application to read the user's information about their encounters with health care practitioners, including things like location, time of appointment, and name of organization the visit was with.
**String** (/reference/java/lang/String)	**READ_MEDICAL_DATA_VITAL_SIGNS** (/reference/android/health/connect/HealthPermissions#READ_MEDICAL_DATA_VITAL_SIGNS)<br><br>Allows an application to read the user's vital signs data.
**String** (/reference/java/lang/String)	**READ_MENSTRUATION** (/reference/android/health/connect/HealthPermissions#READ_MENSTRUATION)<br><br>Allows an application to read the user's menstruation data.
**String** (/reference/java/lang/String)	**READ_MINDFULNESS** (/reference/android/health/connect/HealthPermissions#READ_MINDFULNESS)<br><br>Allows an application to read user's mindfulness data.
**String** (/reference/java/lang/String)	**READ_NUTRITION** (/reference/android/health/connect/HealthPermissions#READ_NUTRITION)<br><br>Allows an application to read the user's nutrition data.
**String** (/reference/java/lang/String)	**READ_OVULATION_TEST** (/reference/android/health/connect/HealthPermissions#READ_OVULATION_TEST)<br><br>Allows an application to read the user's ovulation test data.

String (/reference/java/lang/String)	READ_OXYGEN_SATURATION (/reference/android/health/connect/HealthPermissions#READ_OXYGEN_SATURATION)<br><br>Allows an application to read the user's oxygen saturation data.
String (/reference/java/lang/String)	READ_PLANNED_EXERCISE (/reference/android/health/connect/HealthPermissions#READ_PLANNED_EXERCISE)<br><br>Allows an application to read the user's training plan data.
String (/reference/java/lang/String)	READ_POWER (/reference/android/health/connect/HealthPermissions#READ_POWER)<br><br>Allows an application to read the user's power data.
String (/reference/java/lang/String)	READ_RESPIRATORY_RATE (/reference/android/health/connect/HealthPermissions#READ_RESPIRATORY_RATE)<br><br>Allows an application to read the user's respiratory rate data.
String (/reference/java/lang/String)	READ_RESTING_HEART_RATE (/reference/android/health/connect/HealthPermissions#READ_RESTING_HEART_RATE)<br><br>Allows an application to read the user's resting heart rate data.
String (/reference/java/lang/String)	READ_SEXUAL_ACTIVITY (/reference/android/health/connect/HealthPermissions#READ_SEXUAL_ACTIVITY)<br><br>Allows an application to read the user's sexual activity data.
String (/reference/java/lang/String)	READ_SKIN_TEMPERATURE (/reference/android/health/connect/HealthPermissions#READ_SKIN_TEMPERATURE)<br><br>Allows an application to read the user's skin temperature data.

`String` (/reference/java/lang/String)	`READ_SLEEP` (/reference/android/health/connect/HealthPermissions#READ_SLEEP)<br><br>Allows an application to read the user's sleep data.
`String` (/reference/java/lang/String)	`READ_SPEED` (/reference/android/health/connect/HealthPermissions#READ_SPEED)<br><br>Allows an application to read the user's speed data.
`String` (/reference/java/lang/String)	`READ_STEPS` (/reference/android/health/connect/HealthPermissions#READ_STEPS)<br><br>Allows an application to read the user's steps data.
`String` (/reference/java/lang/String)	`READ_TOTAL_CALORIES_BURNED` (/reference/android/health/connect/HealthPermissions#READ_TOTAL_CALORIES_BURNED)<br><br>Allows an application to read the user's total calories burned data.
`String` (/reference/java/lang/String)	`READ_VO2_MAX` (/reference/android/health/connect/HealthPermissions#READ_VO2_MAX)<br><br>Allows an application to read the user's vo2 maximum data.
`String` (/reference/java/lang/String)	`READ_WEIGHT` (/reference/android/health/connect/HealthPermissions#READ_WEIGHT)<br><br>Allows an application to read the user's weight data.
`String` (/reference/java/lang/String)	`READ_WHEELCHAIR_PUSHES` (/reference/android/health/connect/HealthPermissions#READ_WHEELCHAIR_PUSHES)<br><br>Allows an application to read the user's wheelchair pushes data.
`String` (/reference/java/lang/String)	`START_ONBOARDING` (/reference/android/health/connect/HealthPermissions#START_ONBOARDING)

	Allows an application to launch client onboarding activities responsible for connecting to Health Connect.
`String` (/reference/java/lang/String)	`WRITE_ACTIVE_CALORIES_BURNED` (/reference/android/health/connect/HealthPermissions#WRITE_ACTIVE_CALORIES_BURNED)<br><br>Allows an application to write the user's calories burned data.
`String` (/reference/java/lang/String)	`WRITE_ACTIVITY_INTENSITY` (/reference/android/health/connect/HealthPermissions#WRITE_ACTIVITY_INTENSITY)<br><br>Allows an application to write the user's activity intensity data.
`String` (/reference/java/lang/String)	`WRITE_BASAL_BODY_TEMPERATURE` (/reference/android/health/connect/HealthPermissions#WRITE_BASAL_BODY_TEMPERATURE)<br><br>Allows an application to write the user's basal body temperature data.
`String` (/reference/java/lang/String)	`WRITE_BASAL_METABOLIC_RATE` (/reference/android/health/connect/HealthPermissions#WRITE_BASAL_METABOLIC_RATE)<br><br>Allows an application to write the user's basal metabolic rate data.
`String` (/reference/java/lang/String)	`WRITE_BLOOD_GLUCOSE` (/reference/android/health/connect/HealthPermissions#WRITE_BLOOD_GLUCOSE)<br><br>Allows an application to write the user's blood glucose data.
`String` (/reference/java/lang/String)	`WRITE_BLOOD_PRESSURE` (/reference/android/health/connect/HealthPermissions#WRITE_BLOOD_PRESSURE)<br><br>Allows an application to write the user's blood pressure data.
`String` (/reference/java/lang/String)	`WRITE_BODY_FAT`

	(/reference/android/health/connect/HealthPermissions#WRITE_BODY_FAT)<br><br>Allows an application to write the user's body fat data.
`String` (/reference/java/lang/String)	`WRITE_BODY_TEMPERATURE` (/reference/android/health/connect/HealthPermissions#WRITE_BODY_TEMPERATURE)<br><br>Allows an application to write the user's body temperature data.
`String` (/reference/java/lang/String)	`WRITE_BODY_WATER_MASS` (/reference/android/health/connect/HealthPermissions#WRITE_BODY_WATER_MASS)<br><br>Allows an application to write the user's body water mass data.
`String` (/reference/java/lang/String)	`WRITE_BONE_MASS` (/reference/android/health/connect/HealthPermissions#WRITE_BONE_MASS)<br><br>Allows an application to write the user's bone mass data.
`String` (/reference/java/lang/String)	`WRITE_CERVICAL_MUCUS` (/reference/android/health/connect/HealthPermissions#WRITE_CERVICAL_MUCUS)<br><br>Allows an application to write the user's cervical mucus data.
`String` (/reference/java/lang/String)	`WRITE_DISTANCE` (/reference/android/health/connect/HealthPermissions#WRITE_DISTANCE)<br><br>Allows an application to write the user's distance data.
`String` (/reference/java/lang/String)	`WRITE_ELEVATION_GAINED` (/reference/android/health/connect/HealthPermissions#WRITE_ELEVATION_GAINED)<br><br>Allows an application to write the user's elevation gained data.
`String`	`WRITE_EXERCISE`

(/reference/java/lang/String)	(/reference/android/health/connect/HealthPermissions#WRITE_EXERCISE)<br><br>Allows an application to write the user's exercise data.
`String` (/reference/java/lang/String)	`WRITE_EXERCISE_ROUTE` (/reference/android/health/connect/HealthPermissions#WRITE_EXERCISE_ROUTE)<br><br>Allows an application to write the user's exercise route.
`String` (/reference/java/lang/String)	`WRITE_FLOORS_CLIMBED` (/reference/android/health/connect/HealthPermissions#WRITE_FLOORS_CLIMBED)<br><br>Allows an application to write the user's floors climbed data.
`String` (/reference/java/lang/String)	`WRITE_HEART_RATE` (/reference/android/health/connect/HealthPermissions#WRITE_HEART_RATE)<br><br>Allows an application to write the user's heart rate data.
`String` (/reference/java/lang/String)	`WRITE_HEART_RATE_VARIABILITY` (/reference/android/health/connect/HealthPermissions#WRITE_HEART_RATE_VARIABILITY)<br><br>Allows an application to write the user's heart rate variability data.
`String` (/reference/java/lang/String)	`WRITE_HEIGHT` (/reference/android/health/connect/HealthPermissions#WRITE_HEIGHT)<br><br>Allows an application to write the user's height data.
`String` (/reference/java/lang/String)	`WRITE_HYDRATION` (/reference/android/health/connect/HealthPermissions#WRITE_HYDRATION)<br><br>Allows an application to write the user's hydration data.
`String`	`WRITE_INTERMENSTRUAL_BLEEDING`

(/reference/java/lang/String)	(/reference/android/health/connect/HealthPermissions#WRITE_INTERMENSTRUAL_BLEEDING)<br><br>Allows an application to write the user's intermenstrual bleeding data.
**String** (/reference/java/lang/String)	**WRITE_LEAN_BODY_MASS** (/reference/android/health/connect/HealthPermissions#WRITE_LEAN_BODY_MASS)<br><br>Allows an application to write the user's lean body mass data.
**String** (/reference/java/lang/String)	**WRITE_MEDICAL_DATA** (/reference/android/health/connect/HealthPermissions#WRITE_MEDICAL_DATA)<br><br>Allows an application to write the user's medical data.
**String** (/reference/java/lang/String)	**WRITE_MENSTRUATION** (/reference/android/health/connect/HealthPermissions#WRITE_MENSTRUATION)<br><br>Allows an application to write the user's menstruation data.
**String** (/reference/java/lang/String)	**WRITE_MINDFULNESS** (/reference/android/health/connect/HealthPermissions#WRITE_MINDFULNESS)<br><br>Allows an application to write user's mindfulness data.
**String** (/reference/java/lang/String)	**WRITE_NUTRITION** (/reference/android/health/connect/HealthPermissions#WRITE_NUTRITION)<br><br>Allows an application to write the user's nutrition data.
**String** (/reference/java/lang/String)	**WRITE_OVULATION_TEST** (/reference/android/health/connect/HealthPermissions#WRITE_OVULATION_TEST)<br><br>Allows an application to write the user's ovulation test data.
**String**	**WRITE_OXYGEN_SATURATION**

(/reference/java/lang/String)	(/reference/android/health/connect/HealthPermissions#WRITE_OXYGEN_SATURATION)<br><br>Allows an application to write the user's oxygen saturation data.
**String** (/reference/java/lang/String)	**WRITE_PLANNED_EXERCISE** (/reference/android/health/connect/HealthPermissions#WRITE_PLANNED_EXERCISE)<br><br>Allows an application to write the user's training plan data.
**String** (/reference/java/lang/String)	**WRITE_POWER** (/reference/android/health/connect/HealthPermissions#WRITE_POWER)<br><br>Allows an application to write the user's power data.
**String** (/reference/java/lang/String)	**WRITE_RESPIRATORY_RATE** (/reference/android/health/connect/HealthPermissions#WRITE_RESPIRATORY_RATE)<br><br>Allows an application to write the user's respiratory rate data.
**String** (/reference/java/lang/String)	**WRITE_RESTING_HEART_RATE** (/reference/android/health/connect/HealthPermissions#WRITE_RESTING_HEART_RATE)<br><br>Allows an application to write the user's resting heart rate data.
**String** (/reference/java/lang/String)	**WRITE_SEXUAL_ACTIVITY** (/reference/android/health/connect/HealthPermissions#WRITE_SEXUAL_ACTIVITY)<br><br>Allows an application to write the user's sexual activity data.
**String** (/reference/java/lang/String)	**WRITE_SKIN_TEMPERATURE** (/reference/android/health/connect/HealthPermissions#WRITE_SKIN_TEMPERATURE)<br><br>Allows an application to write the user's skin temperature data.
**String** (/reference/java/lang/String)	**WRITE_SLEEP** (/reference/android/health/connect/HealthPermissions#WRITE_SLEEP)

	Allows an application to write the user's sleep data.
`String` (/reference/java/lang/String)	`WRITE_SPEED` (/reference/android/health/connect/HealthPermissions#WRITE_SPEED)<br>Allows an application to write the user's speed data.
`String` (/reference/java/lang/String)	`WRITE_STEPS` (/reference/android/health/connect/HealthPermissions#WRITE_STEPS)<br>Allows an application to write the user's steps data.
`String` (/reference/java/lang/String)	`WRITE_TOTAL_CALORIES_BURNED` (/reference/android/health/connect/HealthPermissions#WRITE_TOTAL_CALORIES_BURNED)<br>Allows an application to write the user's total calories burned data.
`String` (/reference/java/lang/String)	`WRITE_VO2_MAX` (/reference/android/health/connect/HealthPermissions#WRITE_VO2_MAX)<br>Allows an application to write the user's vo2 maximum data.
`String` (/reference/java/lang/String)	`WRITE_WEIGHT` (/reference/android/health/connect/HealthPermissions#WRITE_WEIGHT)<br>Allows an application to write the user's weight data.
`String` (/reference/java/lang/String)	`WRITE_WHEELCHAIR_PUSHES` (/reference/android/health/connect/HealthPermissions#WRITE_WHEELCHAIR_PUSHES)<br>Allows an application to write the user's wheelchair pushes data.

Inherited methods

From class `java.lang.Object` (/reference/java/lang/Object)

`Object` (/reference/java/lang/Object)	`clone` (/reference/java/lang/Object#clone())`()`<br>Creates and returns a copy of this object.
`boolean`	`equals` (/reference/java/lang/Object#equals(java.lang.Object))`(Object` (/reference/java/lang/Object) `obj)`<br>Indicates whether some other object is "equal to" this one.
`void`	`finalize` (/reference/java/lang/Object#finalize())`()`<br>Called by the garbage collector on an object when garbage collection determines that there are no more references to the object.
`final Class` (/reference/java/lang/Class)`<?>`	`getClass` (/reference/java/lang/Object#getClass())`()`<br>Returns the runtime class of this `Object`.
`int`	`hashCode` (/reference/java/lang/Object#hashCode())`()`<br>Returns a hash code value for the object.
`final void`	`notify` (/reference/java/lang/Object#notify())`()`<br>Wakes up a single thread that is waiting on this object's monitor.
`final void`	`notifyAll` (/reference/java/lang/Object#notifyAll())`()`<br>Wakes up all threads that are waiting on this object's monitor.
`String` (/reference/java/lang/String)	`toString` (/reference/java/lang/Object#toString())`()`<br>Returns a string representation of the object.

`final void`	`wait` (/reference/java/lang/Object#wait(long,%20int)) `(long timeoutMillis, int nanos)`<br><br>Causes the current thread to wait until it is awakened, typically by being *notified* or *interrupted*, or until a certain amount of real time has elapsed.
`final void`	`wait` (/reference/java/lang/Object#wait(long)) `(long timeoutMillis)`<br><br>Causes the current thread to wait until it is awakened, typically by being *notified* or *interrupted*, or until a certain amount of real time has elapsed.
`final void`	`wait` (/reference/java/lang/Object#wait()) `()`<br><br>Causes the current thread to wait until it is awakened, typically by being *notified* or *interrupted*.

Constants

READ_ACTIVE_CALORIES_BURNED

Added in API level 34 (/guide/topics/manifest/uses-sdk-element#ApiLevels)

```
public static final String (/reference/java/lang/String) READ_ACTIVE_CALORIES_BURNED
```

Allows an application to read the user's active calories burned data.

Protection level: dangerous.

Constant Value: "android.permission.health.READ_ACTIVE_CALORIES_BURNED"

READ_ACTIVITY_INTENSITY

Added in API level 36 (/guide/topics/manifest/uses-sdk-element#ApiLevels)

Also in U Extensions 16 (/sdkExtensions)

```
public static final String (/reference/java/lang/String) READ_ACTIVITY_INTENSITY
```

Allows an application to read the user's activity intensity data.

Protection level: dangerous.

Constant Value: "android.permission.health.READ_ACTIVITY_INTENSITY"

READ_BASAL_BODY_TEMPERATURE

Added in API level 34 (/guide/topics/manifest/uses-sdk-element#ApiLevels)

```
public static final String (/reference/java/lang/String) READ_BASAL_BODY_TEMPERATURE
```

Allows an application to read the user's body temperature data.

Protection level: dangerous.

Constant Value: "android.permission.health.READ_BASAL_BODY_TEMPERATURE"

READ_BASAL_METABOLIC_RATE

Added in API level 34 (/guide/topics/manifest/uses-sdk-element#ApiLevels)

```
public static final String (/reference/java/lang/String) READ_BASAL_METABOLIC_RATE
```

Allows an application to read the user's basal metabolic rate data.

Protection level: dangerous.

Constant Value: "android.permission.health.READ_BASAL_METABOLIC_RATE"

READ_BLOOD_GLUCOSE

Added in API level 34 (/guide/topics/manifest/uses-sdk-element#ApiLevels)

```
public static final String (/reference/java/lang/String) READ_BLOOD_GLUCOSE
```

Allows an application to read the user's blood glucose data.

Protection level: dangerous.

Constant Value: "android.permission.health.READ_BLOOD_GLUCOSE"

READ_BLOOD_PRESSURE

Added in API level 34 (/guide/topics/manifest/uses-sdk-element#ApiLevels)

```
public static final String (/reference/java/lang/String) READ_BLOOD_PRESSURE
```

Allows an application to read the user's blood pressure data.

Protection level: dangerous.

Constant Value: "android.permission.health.READ_BLOOD_PRESSURE"

READ_BODY_FAT

Added in API level 34 (/guide/topics/manifest/uses-sdk-element#ApiLevels)

```
public static final String (/reference/java/lang/String) READ_BODY_FAT
```

Allows an application to read the user's body fat data.

Protection level: dangerous.

Constant Value: "android.permission.health.READ_BODY_FAT"

READ_BODY_TEMPERATURE

Added in API level 34 (/guide/topics/manifest/uses-sdk-element#ApiLevels)

```
public static final String (/reference/java/lang/String) READ_BODY_TEMPERATURE
```

Allows an application to read the user's body temperature data.

Protection level: dangerous.

Constant Value: "android.permission.health.READ_BODY_TEMPERATURE"

READ_BODY_WATER_MASS

Added in API level 34 (/guide/topics/manifest/uses-sdk-element#ApiLevels)

```
public static final String (/reference/java/lang/String) READ_BODY_WATER_MASS
```

Allows an application to read the user's body water mass data.

Protection level: dangerous.

Constant Value: "android.permission.health.READ_BODY_WATER_MASS"

READ_BONE_MASS

Added in API level 34 (/guide/topics/manifest/uses-sdk-element#ApiLevels)

```
public static final String (/reference/java/lang/String) READ_BONE_MASS
```

Allows an application to read the user's bone mass data.

Protection level: dangerous.

Constant Value: "android.permission.health.READ_BONE_MASS"

READ_CERVICAL_MUCUS

Added in API level 34 (/guide/topics/manifest/uses-sdk-element#ApiLevels)

```
public static final String (/reference/java/lang/String) READ_CERVICAL_MUCUS
```

Allows an application to read the user's cervical mucus data.

Protection level: dangerous.

Constant Value: "android.permission.health.READ_CERVICAL_MUCUS"

READ_DISTANCE

Added in API level 34 (/guide/topics/manifest/uses-sdk-element#ApiLevels)

```
public static final String (/reference/java/lang/String) READ_DISTANCE
```

Allows an application to read the user's distance data.

Protection level: dangerous.

Constant Value: "android.permission.health.READ_DISTANCE"

READ_ELEVATION_GAINED

Added in API level 34 (/guide/topics/manifest/uses-sdk-element#ApiLevels)

```
public static final String (/reference/java/lang/String) READ_ELEVATION_GAINED
```

Allows an application to read the user's elevation gained data.

Protection level: dangerous.

Constant Value: "android.permission.health.READ_ELEVATION_GAINED"

READ_EXERCISE

Added in API level 34 (/guide/topics/manifest/uses-sdk-element#ApiLevels)

```
public static final String (/reference/java/lang/String) READ_EXERCISE
```

Allows an application to read the user's exercise data.

Protection level: dangerous.

Constant Value: "android.permission.health.READ_EXERCISE"

READ_EXERCISE_ROUTES

Added in API level 35 (/guide/topics/manifest/uses-sdk-element#ApiLevels)
Also in U Extensions 12 (/sdkExtensions)

```
public static final String (/reference/java/lang/String) READ_EXERCISE_ROUTES
```

Allows an application to read `ExerciseRoute` (/reference/android/health/connect/datatypes/ExerciseRoute).

This permission can only be granted manually by a user in Health Connect settings or in the route request activity which can be launched using `ERROR(/ACTION_REQUEST_EXERCISE_ROUTE)` (/). Attempts to request the permission by applications

will be ignored.

Applications should check if the permission has been granted before reading `ExerciseRoute` (/reference/android/health/connect/datatypes/ExerciseRoute).

Protection level: dangerous.

Constant Value: "android.permission.health.READ_EXERCISE_ROUTES"

READ_FLOORS_CLIMBED

Added in API level 34 (/guide/topics/manifest/uses-sdk-element#ApiLevels)

```
public static final String (/reference/java/lang/String) READ_FLOORS_CLIMBED
```

Allows an application to read the user's floors climbed data.

Protection level: dangerous.

Constant Value: "android.permission.health.READ_FLOORS_CLIMBED"

READ_HEALTH_DATA_HISTORY

Added in API level 35 (/guide/topics/manifest/uses-sdk-element#ApiLevels)
Also in U Extensions 13 (/sdkExtensions)

```
public static final String (/reference/java/lang/String) READ_HEALTH_DATA_HISTORY
```

Allows an application to read the entire history of health data (of any type).

Protection level: dangerous.

Constant Value: "android.permission.health.READ_HEALTH_DATA_HISTORY"

READ_HEALTH_DATA_IN_BACKGROUND

[illegible]

```
public static final Stri
```

Allows an application to read health data (of any type) in background.

Protection level: dangerous.

Constant Value: "android.permission.health.READ_HEALTH_DATA_IN_BACKGROUND"

READ_HEART_RATE

Added in API level 34 (/guide/topics/manifest/uses-sdk-element#ApiLevels)

```
public static final String (/reference/java/lang/String) READ_HEART_RATE
```

Allows an application to read the user's heart rate data.

Protection level: dangerous.

Constant Value: "android.permission.health.READ_HEART_RATE"

READ_HEART_RATE_VARIABILITY

Added in API level 34 (/guide/topics/manifest/uses-sdk-element#ApiLevels)

```
public static final String (/reference/java/lang/String) READ_HEART_RATE_VARIABILITY
```

Allows an application to read the user's heart rate variability data.

Protection level: dangerous.

Constant Value: "android.permission.health.READ_HEART_RATE_VARIABILITY"

READ_HEIGHT

Added in API level 34 (/guide/topics/manifest/uses-sdk-element#ApiLevels)

```
public static final String (/reference/java/lang/String) READ_HEIGHT
```

Allows an application to read the user's height data.

Protection level: dangerous.

Constant Value: "android.permission.health.READ_HEIGHT"

READ_HYDRATION

Added in API level 34 (/guide/topics/manifest/uses-sdk-element#ApiLevels)

```
public static final String (/reference/java/lang/String) READ_HYDRATION
```

Allows an application to read the user's hydration data.

Protection level: dangerous.

Constant Value: "android.permission.health.READ_HYDRATION"

READ_INTERMENSTRUAL_BLEEDING

Added in API level 34 (/guide/topics/manifest/uses-sdk-element#ApiLevels)

```
public static final String (/reference/java/lang/String) READ_INTERMENSTRUAL_BLEEDING
```

Allows an application to read the user's intermenstrual bleeding data.

Protection level: dangerous.

Constant Value: "android.permission.health.READ_INTERMENSTRUAL_BLEEDING"

READ_LEAN_BODY_MASS

Added in API level 34 (/guide/topics/manifest/uses-sdk-element#ApiLevels)

```
public static final String (/reference/java/lang/String) READ_LEAN_BODY_MASS
```

Allows an application to read the user's lean body mass data.

Protection level: dangerous.

Constant Value: "android.permission.health.READ_LEAN_BODY_MASS"

READ_MEDICAL_DATA_ALLERGIES_INTOLERANCES

Added in API level 36 (/guide/topics/manifest/uses-sdk-element#ApiLevels)
Also in U Extensions 16 (/sdkExtensions)

```
public static final String (/reference/java/lang/String) READ_MEDICAL_DATA_ALLERGIES_INT
```

Allows an application to read the user's data about allergies and intolerances.

Protection level: dangerous.

Constant Value:
"android.permission.health.READ_MEDICAL_DATA_ALLERGIES_INTOLERANCES"

READ_MEDICAL_DATA_CONDITIONS

Added in API level 36 (/guide/topics/manifest/uses-sdk-element#ApiLevels)
Also in U Extensions 16 (/sdkExtensions)

```
public static final String (/reference/java/lang/String) READ_MEDICAL_DATA_CONDITIONS
```

Allows an application to read the user's data about medical conditions.

Protection level: dangerous.

Constant Value: "android.permission.health.READ_MEDICAL_DATA_CONDITIONS"

READ_MEDICAL_DATA_LABORATORY_RESULTS

Added in API level 36 (/guide/topics/manifest/uses-sdk-element#ApiLevels)
Also in U Extensions 16 (/sdkExtensions)

```
public static final String (/reference/java/lang/String) READ_MEDICAL_DATA_LABORATORY_RES
```

Allows an application to read the user's laboratory result data.

Protection level: dangerous.

Constant Value: "android.permission.health.READ_MEDICAL_DATA_LABORATORY_RESULTS"

READ_MEDICAL_DATA_MEDICATIONS

Added in API level 36 (/guide/topics/manifest/uses-sdk-element#ApiLevels)
Also in U Extensions 16 (/sdkExtensions)

```
public static final String (/reference/java/lang/String) READ_MEDICAL_DATA_MEDICATIONS
```

Allows an application to read the user's medication data.

Protection level: dangerous.

Constant Value: "android.permission.health.READ_MEDICAL_DATA_MEDICATIONS"

READ_MEDICAL_DATA_PERSONAL_DETAILS

Added in API level 36 (/guide/topics/manifest/uses-sdk-element#ApiLevels)
Also in U Extensions 16 (/sdkExtensions)

```
public static final String (/reference/java/lang/String) READ_MEDICAL_DATA_PERSONAL_DETAI
```

Allows an application to read the user's personal details.

This is demographic information such as name, date of birth, contact details like address or telephone number and so on. For more examples see the FHIR Patient resource (https://www.hl7.org/fhir/patient.html).

Protection level: dangerous.

Constant Value: "android.permission.health.READ_MEDICAL_DATA_PERSONAL_DETAILS"

READ_MEDICAL_DATA_PRACTITIONER_DETAILS

Added in API level 36 (/guide/topics/manifest/uses-sdk-element#ApiLevels)
Also in U Extensions 16 (/sdkExtensions)

```
public static final String (/reference/java/lang/String) READ_MEDICAL_DATA_PRACTITIONER_I
```

Allows an application to read the user's data about the practitioners who have interacted with them in their medical record. This is the information about the clinicians (doctors, nurses, etc) but also other practitioners (masseurs, physiotherapists, etc) who have been involved with the patient.

Protection level: dangerous.

Constant Value: "android.permission.health.READ_MEDICAL_DATA_PRACTITIONER_DETAILS"

READ_MEDICAL_DATA_PREGNANCY

Added in API level 36 (/guide/topics/manifest/uses-sdk-element#ApiLevels)
Also in U Extensions 16 (/sdkExtensions)

```
public static final String (/reference/java/lang/String) READ_MEDICAL_DATA_PREGNANCY
```

Allows an application to read the user's pregnancy data.

Protection level: dangerous.

Constant Value: "android.permission.health.READ_MEDICAL_DATA_PREGNANCY"

READ_MEDICAL_DATA_PROCEDURES

Added in API level 36 (/guide/topics/manifest/uses-sdk-element#ApiLevels)
Also in U Extensions 16 (/sdkExtensions)

```
public static final String (/reference/java/lang/String) READ_MEDICAL_DATA_PROCEDURES
```

Allows an application to read the user's data about medical procedures.

Protection level: dangerous.

Constant Value: "android.permission.health.READ_MEDICAL_DATA_PROCEDURES"

READ_MEDICAL_DATA_SOCIAL_HISTORY

Added in API level 36 (/guide/topics/manifest/uses-sdk-element#ApiLevels)
Also in U Extensions 16 (/sdkExtensions)

```
public static final String (/reference/java/lang/String) READ_MEDICAL_DATA_SOCIAL_HISTORY
```

Allows an application to read the user's social history data.

Protection level: dangerous.

Constant Value: "android.permission.health.READ_MEDICAL_DATA_SOCIAL_HISTORY"

READ_MEDICAL_DATA_VACCINES

Added in API level 36 (/guide/topics/manifest/uses-sdk-element#ApiLevels)
Also in U Extensions 16 (/sdkExtensions)

```
public static final String (/reference/java/lang/String) READ_MEDICAL_DATA_VACCINES
```

Allows an application to read the user's data about immunizations and vaccinations.

Protection level: dangerous.

Constant Value: "android.permission.health.READ_MEDICAL_DATA_VACCINES"

READ_MEDICAL_DATA_VISITS

Added in API level 36 (/guide/topics/manifest/uses-sdk-element#ApiLevels)
Also in U Extensions 16 (/sdkExtensions)

```
public static final String (/reference/java/lang/String) READ_MEDICAL_DATA_VISITS
```

Allows an application to read the user's information about their encounters with health care practitioners, including things like location, time of appointment, and name of organization the visit was with. Despite the name visit it covers remote encounters such as telephone or videoconference appointments.

Protection level: dangerous.

Constant Value: "android.permission.health.READ_MEDICAL_DATA_VISITS"

READ_MEDICAL_DATA_VITAL_SIGNS

Added in API level 36 (/guide/topics/manifest/uses-sdk-element#ApiLevels)
Also in U Extensions 16 (/sdkExtensions)

```
public static final String (/reference/java/lang/String) READ_MEDICAL_DATA_VITAL_SIGNS
```

Allows an application to read the user's vital signs data.

Protection level: dangerous.

Constant Value: "android.permission.health.READ_MEDICAL_DATA_VITAL_SIGNS"

READ_MENSTRUATION

Added in API level 34 (/guide/topics/manifest/uses-sdk-element#ApiLevels)

```
public static final String (/reference/java/lang/String) READ_MENSTRUATION
```

Allows an application to read the user's menstruation data.

Protection level: dangerous.

Constant Value: "android.permission.health.READ_MENSTRUATION"

READ_MINDFULNESS

Added in API level 36 (/guide/topics/manifest/uses-sdk-element#ApiLevels)
Also in U Extensions 15 (/sdkExtensions)

```
public static final String (/reference/java/lang/String) READ_MINDFULNESS
```

Allows an application to read user's mindfulness data.

Protection level: dangerous.

Constant Value: "android.permission.health.READ_MINDFULNESS"

READ_NUTRITION

Added in API level 34 (/guide/topics/manifest/uses-sdk-element#ApiLevels)

```
public static final String (/reference/java/lang/String) READ_NUTRITION
```

Allows an application to read the user's nutrition data.

Protection level: dangerous.

Constant Value: "android.permission.health.READ_NUTRITION"

READ_OVULATION_TEST

Added in API level 34 (/guide/topics/manifest/uses-sdk-element#ApiLevels)

```
public static final String (/reference/java/lang/String) READ_OVULATION_TEST
```

Allows an application to read the user's ovulation test data.

Protection level: dangerous.

Constant Value: "android.permission.health.READ_OVULATION_TEST"

READ_OXYGEN_SATURATION

Added in API level 34 (/guide/topics/manifest/uses-sdk-element#ApiLevels)

```
public static final String (/reference/java/lang/String) READ_OXYGEN_SATURATION
```

Allows an application to read the user's oxygen saturation data.

Protection level: dangerous.

Constant Value: "android.permission.health.READ_OXYGEN_SATURATION"

READ_PLANNED_EXERCISE

Added in API level 35 (/guide/topics/manifest/uses-sdk-element#ApiLevels)
Also in U Extensions 13 (/sdkExtensions)

```
public static final String (/reference/java/lang/String) READ_PLANNED_EXERCISE
```

Allows an application to read the user's training plan data.

Protection level: dangerous.

Constant Value: "android.permission.health.READ_PLANNED_EXERCISE"

READ_POWER

Added in API level 34 (/guide/topics/manifest/uses-sdk-element#ApiLevels)

```
public static final String (/reference/java/lang/String) READ_POWER
```

Allows an application to read the user's power data.

Protection level: dangerous.

Constant Value: "android.permission.health.READ_POWER"

READ_RESPIRATORY_RATE

Added in API level 34 (/guide/topics/manifest/uses-sdk-element#ApiLevels)

```
public static final String (/reference/java/lang/String) READ_RESPIRATORY_RATE
```

Allows an application to read the user's respiratory rate data.

Protection level: dangerous.

Constant Value: "android.permission.health.READ_RESPIRATORY_RATE"

READ_RESTING_HEART_RATE

Added in API level 34 (/guide/topics/manifest/uses-sdk-element#ApiLevels)

```
public static final String (/reference/java/lang/String) READ_RESTING_HEART_RATE
```

Allows an application to read the user's resting heart rate data.

Protection level: dangerous.

Constant Value: "android.permission.health.READ_RESTING_HEART_RATE"

READ_SEXUAL_ACTIVITY

Added in API level 34 (/guide/topics/manifest/uses-sdk-element#ApiLevels)

```
public static final String (/reference/java/lang/String) READ_SEXUAL_ACTIVITY
```

Allows an application to read the user's sexual activity data.

Protection level: dangerous.

Constant Value: "android.permission.health.READ_SEXUAL_ACTIVITY"

READ_SKIN_TEMPERATURE

Added in API level 35 (/guide/topics/manifest/uses-sdk-element#ApiLevels)
Also in U Extensions 13 (/sdkExtensions)

```
public static final String (/reference/java/lang/String) READ_SKIN_TEMPERATURE
```

Allows an application to read the user's skin temperature data.

Protection level: dangerous.

Constant Value: "android.permission.health.READ_SKIN_TEMPERATURE"

READ_SLEEP

Added in API level 34 (/guide/topics/manifest/uses-sdk-element#ApiLevels)

```
public static final String (/reference/java/lang/String) READ_SLEEP
```

Allows an application to read the user's sleep data.

Protection level: dangerous.

Constant Value: "android.permission.health.READ_SLEEP"

READ_SPEED

Added in API level 34 (/guide/topics/manifest/uses-sdk-element#ApiLevels)

```
public static final String (/reference/java/lang/String) READ_SPEED
```

Allows an application to read the user's speed data.

Protection level: dangerous.

Constant Value: "android.permission.health.READ_SPEED"

READ_STEPS

Added in API level 34 (/guide/topics/manifest/uses-sdk-element#ApiLevels)

```
public static final String (/reference/java/lang/String) READ_STEPS
```

Allows an application to read the user's steps data.

Protection level: dangerous.

Constant Value: "android.permission.health.READ_STEPS"

READ_TOTAL_CALORIES_BURNED

Added in API level 34 (/guide/topics/manifest/uses-sdk-element#ApiLevels)

```
public static final String (/reference/java/lang/String) READ_TOTAL_CALORIES_BURNED
```

Allows an application to read the user's total calories burned data.

Protection level: dangerous.

Constant Value: "android.permission.health.READ_TOTAL_CALORIES_BURNED"

READ_VO2_MAX

Added in API level 34 (/guide/topics/manifest/uses-sdk-element#ApiLevels)

```
public static final String (/reference/java/lang/String) READ_VO2_MAX
```

Allows an application to read the user's vo2 maximum data.

Protection level: dangerous.

Constant Value: "android.permission.health.READ_VO2_MAX"

READ_WEIGHT

Added in API level 34 (/guide/topics/manifest/uses-sdk-element#ApiLevels)

```
public static final String (/reference/java/lang/String) READ_WEIGHT
```

Allows an application to read the user's weight data.

Protection level: dangerous.

Constant Value: "android.permission.health.READ_WEIGHT"

READ_WHEELCHAIR_PUSHES

Added in API level 34 (/guide/topics/manifest/uses-sdk-element#ApiLevels)

```
public static final String (/reference/java/lang/String) READ_WHEELCHAIR_PUSHES
```

Allows an application to read the user's wheelchair pushes data.

Protection level: dangerous.

Constant Value: "android.permission.health.READ_WHEELCHAIR_PUSHES"

START_ONBOARDING

Added in version 36.1 (/topic/libraries/support-library/revisions)

```
public static final String (/reference/java/lang/String) START_ONBOARDING
```

Allows an application to launch client onboarding activities responsible for connecting to Health Connect. This permission can only be held by the system. Client apps that choose to export an onboarding activity must guard it with this permission so that only the system can launch it.

See `HealthConnectManager.ACTION_SHOW_ONBOARDING`

(/reference/android/health/connect/HealthConnectManager#ACTION_SHOW_ONBOARDING) for the corresponding intent used by the system to launch onboarding activities.

Protection level: signature.

Constant Value: "android.permission.health.START_ONBOARDING"

WRITE_ACTIVE_CALORIES_BURNED

Added in API level 34 (/guide/topics/manifest/uses-sdk-element#ApiLevels)

```
public static final String (/reference/java/lang/String) WRITE_ACTIVE_CALORIES_BURNED
```

Allows an application to write the user's calories burned data.

Protection level: dangerous.

Constant Value: "android.permission.health.WRITE_ACTIVE_CALORIES_BURNED"

WRITE_ACTIVITY_INTENSITY

Added in API level 36 (/guide/topics/manifest/uses-sdk-element#ApiLevels)
Also in U Extensions 16 (/sdkExtensions)

```
public static final String (/reference/java/lang/String) WRITE_ACTIVITY_INTENSITY
```

Allows an application to write the user's activity intensity data.

Protection level: dangerous.

Constant Value: "android.permission.health.WRITE_ACTIVITY_INTENSITY"

WRITE_BASAL_BODY_TEMPERATURE

Added in API level 34 (/guide/topics/manifest/uses-sdk-element#ApiLevels)

```
public static final String (/reference/java/lang/String) WRITE_BASAL_BODY_TEMPERATURE
```

Allows an application to write the user's basal body temperature data.

Protection level: dangerous.

Constant Value: "android.permission.health.WRITE_BASAL_BODY_TEMPERATURE"

WRITE_BASAL_METABOLIC_RATE

Added in API level 34 (/guide/topics/manifest/uses-sdk-element#ApiLevels)

```
public static final String (/reference/java/lang/String) WRITE_BASAL_METABOLIC_RATE
```

Allows an application to write the user's basal metabolic rate data.

Protection level: dangerous.

Constant Value: "android.permission.health.WRITE_BASAL_METABOLIC_RATE"

WRITE_BLOOD_GLUCOSE

Added in API level 34 (/guide/topics/manifest/uses-sdk-element#ApiLevels)

```
public static final String (/reference/java/lang/String) WRITE_BLOOD_GLUCOSE
```

Allows an application to write the user's blood glucose data.

Protection level: dangerous.

Constant Value: "android.permission.health.WRITE_BLOOD_GLUCOSE"

WRITE_BLOOD_PRESSURE

Added in API level 34 (/guide/topics/manifest/uses-sdk-element#ApiLevels)

```
public static final String (/reference/java/lang/String) WRITE_BLOOD_PRESSURE
```

Allows an application to write the user's blood pressure data.

Protection level: dangerous.

Constant Value: "android.permission.health.WRITE_BLOOD_PRESSURE"

WRITE_BODY_FAT

Added in API level 34 (/guide/topics/manifest/uses-sdk-element#ApiLevels)

```
public static final String (/reference/java/lang/String) WRITE_BODY_FAT
```

Allows an application to write the user's body fat data.

Protection level: dangerous.

Constant Value: "android.permission.health.WRITE_BODY_FAT"

WRITE_BODY_TEMPERATURE

Added in API level 34 (/guide/topics/manifest/uses-sdk-element#ApiLevels)

```
public static final String (/reference/java/lang/String) WRITE_BODY_TEMPERATURE
```

Allows an application to write the user's body temperature data.

Protection level: dangerous.

Constant Value: "android.permission.health.WRITE_BODY_TEMPERATURE"

WRITE_BODY_WATER_MASS

Added in API level 34 (/guide/topics/manifest/uses-sdk-element#ApiLevels)

```
public static final String (/reference/java/lang/String) WRITE_BODY_WATER_MASS
```

Allows an application to write the user's body water mass data.

Protection level: dangerous.

Constant Value: "android.permission.health.WRITE_BODY_WATER_MASS"

WRITE_BONE_MASS

Added in API level 34 (/guide/topics/manifest/uses-sdk-element#ApiLevels)

```
public static final String (/reference/java/lang/String) WRITE_BONE_MASS
```

Allows an application to write the user's bone mass data.

Protection level: dangerous.

Constant Value: "android.permission.health.WRITE_BONE_MASS"

WRITE_CERVICAL_MUCUS

Added in API level 34 (/guide/topics/manifest/uses-sdk-element#ApiLevels)

```
public static final String (/reference/java/lang/String) WRITE_CERVICAL_MUCUS
```

Allows an application to write the user's cervical mucus data.

Protection level: dangerous.

Constant Value: "android.permission.health.WRITE_CERVICAL_MUCUS"

WRITE_DISTANCE

Added in API level 34 (/guide/topics/manifest/uses-sdk-element#ApiLevels)

```
public static final String (/reference/java/lang/String) WRITE_DISTANCE
```

Allows an application to write the user's distance data.

Protection level: dangerous.

Constant Value: "android.permission.health.WRITE_DISTANCE"

WRITE_ELEVATION_GAINED

Added in API level 34 (/guide/topics/manifest/uses-sdk-element#ApiLevels)

```
public static final String (/reference/java/lang/String) WRITE_ELEVATION_GAINED
```

Allows an application to write the user's elevation gained data.

Protection level: dangerous.

Constant Value: "android.permission.health.WRITE_ELEVATION_GAINED"

WRITE_EXERCISE

Added in API level 34 (/guide/topics/manifest/uses-sdk-element#ApiLevels)

```
public static final String (/reference/java/lang/String) WRITE_EXERCISE
```

Allows an application to write the user's exercise data. Additional permission **WRITE_EXERCISE_ROUTE** (/reference/android/health/connect/HealthPermissions#WRITE_EXERCISE_ROUTE) is required to write user's exercise route.

Protection level: dangerous.

Constant Value: "android.permission.health.WRITE_EXERCISE"

WRITE_EXERCISE_ROUTE

Added in API level 34 (/guide/topics/manifest/uses-sdk-element#ApiLevels)

```
public static final String (/reference/java/lang/String) WRITE_EXERCISE_ROUTE
```

Allows an application to write the user's exercise route.

Protection level: dangerous.

Constant Value: "android.permission.health.WRITE_EXERCISE_ROUTE"

WRITE_FLOORS_CLIMBED

Added in API level 34 (/guide/topics/manifest/uses-sdk-element#ApiLevels)

```
public static final String (/reference/java/lang/String) WRITE_FLOORS_CLIMBED
```

Allows an application to write the user's floors climbed data.

Protection level: dangerous.

Constant Value: "android.permission.health.WRITE_FLOORS_CLIMBED"

WRITE_HEART_RATE

Added in API level 34 (/guide/topics/manifest/uses-sdk-element#ApiLevels)

```
public static final String (/reference/java/lang/String) WRITE_HEART_RATE
```

Allows an application to write the user's heart rate data.

Protection level: dangerous.

Constant Value: "android.permission.health.WRITE_HEART_RATE"

WRITE_HEART_RATE_VARIABILITY

Added in API level 34 (/guide/topics/manifest/uses-sdk-element#ApiLevels)

```
public static final String (/reference/java/lang/String) WRITE_HEART_RATE_VARIABILITY
```

Allows an application to write the user's heart rate variability data.

Protection level: dangerous.

Constant Value: "android.permission.health.WRITE_HEART_RATE_VARIABILITY"

WRITE_HEIGHT

Added in API level 34 (/guide/topics/manifest/uses-sdk-element#ApiLevels)

```
public static final String (/reference/java/lang/String) WRITE_HEIGHT
```

Allows an application to write the user's height data.

Protection level: dangerous.

Constant Value: "android.permission.health.WRITE_HEIGHT"

WRITE_HYDRATION

Added in API level 34 (/guide/topics/manifest/uses-sdk-element#ApiLevels)

```
public static final String (/reference/java/lang/String) WRITE_HYDRATION
```

Allows an application to write the user's hydration data.

Protection level: dangerous.

Constant Value: "android.permission.health.WRITE_HYDRATION"

WRITE_INTERMENSTRUAL_BLEEDING

Added in API level 34 (/guide/topics/manifest/uses-sdk-element#ApiLevels)

```
public static final String (/reference/java/lang/String) WRITE_INTERMENSTRUAL_BLEEDING
```

Allows an application to write the user's intermenstrual bleeding data.

Protection level: dangerous.

Constant Value: "android.permission.health.WRITE_INTERMENSTRUAL_BLEEDING"

WRITE_LEAN_BODY_MASS

Added in API level 34 (/guide/topics/manifest/uses-sdk-element#ApiLevels)

```
public static final String (/reference/java/lang/String) WRITE_LEAN_BODY_MASS
```

Allows an application to write the user's lean body mass data.

Protection level: dangerous.

Constant Value: "android.permission.health.WRITE_LEAN_BODY_MASS"

WRITE_MEDICAL_DATA

Added in API level 36 (/guide/topics/manifest/uses-sdk-element#ApiLevels)
Also in U Extensions 16 (/sdkExtensions)

```
public static final String (/reference/java/lang/String) WRITE_MEDICAL_DATA
```

Allows an application to write the user's medical data.

Protection level: dangerous.

Constant Value: "android.permission.health.WRITE_MEDICAL_DATA"

WRITE_MENSTRUATION

Added in API level 34 (/guide/topics/manifest/uses-sdk-element#ApiLevels)

```
public static final String (/reference/java/lang/String) WRITE_MENSTRUATION
```

Allows an application to write the user's menstruation data.

Protection level: dangerous.

Constant Value: "android.permission.health.WRITE_MENSTRUATION"

WRITE_MINDFULNESS

Added in API level 36 (/guide/topics/manifest/uses-sdk-element#ApiLevels)
Also in U Extensions 15 (/sdkExtensions)

```
public static final String (/reference/java/lang/String) WRITE_MINDFULNESS
```

Allows an application to write user's mindfulness data.

Protection level: dangerous.

Constant Value: "android.permission.health.WRITE_MINDFULNESS"

WRITE_NUTRITION

Added in API level 34 (/guide/topics/manifest/uses-sdk-element#ApiLevels)

```
public static final String (/reference/java/lang/String) WRITE_NUTRITION
```

Allows an application to write the user's nutrition data.

Protection level: dangerous.

Constant Value: "android.permission.health.WRITE_NUTRITION"

WRITE_OVULATION_TEST

Added in API level 34 (/guide/topics/manifest/uses-sdk-element#ApiLevels)

```
public static final String (/reference/java/lang/String) WRITE_OVULATION_TEST
```

Allows an application to write the user's ovulation test data.

Protection level: dangerous.

Constant Value: "android.permission.health.WRITE_OVULATION_TEST"

WRITE_OXYGEN_SATURATION

Added in API level 34 (/guide/topics/manifest/uses-sdk-element#ApiLevels)

```
public static final String (/reference/java/lang/String) WRITE_OXYGEN_SATURATION
```

Allows an application to write the user's oxygen saturation data.

Protection level: dangerous.

Constant Value: "android.permission.health.WRITE_OXYGEN_SATURATION"

WRITE_PLANNED_EXERCISE

Added in API level 35 (/guide/topics/manifest/uses-sdk-element#ApiLevels)
Also in U Extensions 13 (/sdkExtensions)

```
public static final String (/reference/java/lang/String) WRITE_PLANNED_EXERCISE
```

Allows an application to write the user's training plan data.

Protection level: dangerous.

Constant Value: "android.permission.health.WRITE_PLANNED_EXERCISE"

WRITE_POWER

Added in API level 34 (/guide/topics/manifest/uses-sdk-element#ApiLevels)

```
public static final String (/reference/java/lang/String) WRITE_POWER
```

Allows an application to write the user's power data.

Protection level: dangerous.

Constant Value: "android.permission.health.WRITE_POWER"

WRITE_RESPIRATORY_RATE

Added in API level 34 (/guide/topics/manifest/uses-sdk-element#ApiLevels)

```
public static final String (/reference/java/lang/String) WRITE_RESPIRATORY_RATE
```

Allows an application to write the user's respiratory rate data.

Protection level: dangerous.

Constant Value: "android.permission.health.WRITE_RESPIRATORY_RATE"

WRITE_RESTING_HEART_RATE

Added in API level 34 (/guide/topics/manifest/uses-sdk-element#ApiLevels)

```
public static final String (/reference/java/lang/String) WRITE_RESTING_HEART_RATE
```

Allows an application to write the user's resting heart rate data.

Protection level: dangerous.

Constant Value: "android.permission.health.WRITE_RESTING_HEART_RATE"

WRITE_SEXUAL_ACTIVITY

Added in API level 34 (/guide/topics/manifest/uses-sdk-element#ApiLevels)

```
public static final String (/reference/java/lang/String) WRITE_SEXUAL_ACTIVITY
```

Allows an application to write the user's sexual activity data.

Protection level: dangerous.

Constant Value: "android.permission.health.WRITE_SEXUAL_ACTIVITY"

WRITE_SKIN_TEMPERATURE

Added in API level 35 (/guide/topics/manifest/uses-sdk-element#ApiLevels)
Also in U Extensions 13 (/sdkExtensions)

```
public static final String (/reference/java/lang/String) WRITE_SKIN_TEMPERATURE
```

Allows an application to write the user's skin temperature data.

Protection level: dangerous.

Constant Value: "android.permission.health.WRITE_SKIN_TEMPERATURE"

WRITE_SLEEP

Added in API level 34 (/guide/topics/manifest/uses-sdk-element#ApiLevels)

```
public static final String (/reference/java/lang/String) WRITE_SLEEP
```

Allows an application to write the user's sleep data.

Protection level: dangerous.

Constant Value: "android.permission.health.WRITE_SLEEP"

WRITE_SPEED

Added in API level 34 (/guide/topics/manifest/uses-sdk-element#ApiLevels)

```
public static final String (/reference/java/lang/String) WRITE_SPEED
```

Allows an application to write the user's speed data.

Protection level: dangerous.

Constant Value: "android.permission.health.WRITE_SPEED"

WRITE_STEPS

Added in API level 34 (/guide/topics/manifest/uses-sdk-element#ApiLevels)

```
public static final String (/reference/java/lang/String) WRITE_STEPS
```

Allows an application to write the user's steps data.

Protection level: dangerous.

Constant Value: "android.permission.health.WRITE_STEPS"

WRITE_TOTAL_CALORIES_BURNED

Added in API level 34 (/guide/topics/manifest/uses-sdk-element#ApiLevels)

```
public static final String (/reference/java/lang/String) WRITE_TOTAL_CALORIES_BURNED
```

Allows an application to write the user's total calories burned data.

Protection level: dangerous.

Constant Value: "android.permission.health.WRITE_TOTAL_CALORIES_BURNED"

WRITE_VO2_MAX

Added in API level 34 (/guide/topics/manifest/uses-sdk-element#ApiLevels)

```
public static final String (/reference/java/lang/String) WRITE_VO2_MAX
```

Allows an application to write the user's vo2 maximum data.

Protection level: dangerous.

Constant Value: "android.permission.health.WRITE_VO2_MAX"

WRITE_WEIGHT

Added in API level 34 (/guide/topics/manifest/uses-sdk-element#ApiLevels)

```
public static final String (/reference/java/lang/String) WRITE_WEIGHT
```

Allows an application to write the user's weight data.

Protection level: dangerous.

Constant Value: "android.permission.health.WRITE_WEIGHT"

WRITE_WHEELCHAIR_PUSHES

Added in API level 34 (/guide/topics/manifest/uses-sdk-element#ApiLevels)

```
public static final String (/reference/java/lang/String) WRITE_WHEELCHAIR_PUSHES
```

Allows an application to write the user's wheelchair pushes data.

Protection level: dangerous.

Constant Value: "android.permission.health.WRITE_WHEELCHAIR_PUSHES"

Content and code samples on this page are subject to the licenses described in the Content License (/license). Java and OpenJDK are trademarks or registered trademarks of Oracle and/or its affiliates.

Last updated 2025-09-17 UTC.
