# Supplementary material for: On the evolution of security and privacy in Android health APIs
Source: Front Digit Health. 2026 Oct 28;8:1818536. doi: 10.3389/fdgth.2026.1818536 (PMC13751995; doi:10.3389/fdgth.2026.1818536)
Supplement: Supplementary file 1 [file Datasheet1.zip › supplemental-data/documentation-health-connect.pdf]

Starting in 2026, we'll be transitioning away from Google Fit APIs. For more information on the Google Fit migration, see the Migration Guide (/health-and-fitness/guides/health-connect/migrate/migration-guide).

Health Connect: simplify connectivity between apps

Health and fitness apps record valuable data, including medical records. With permission from your users, you can unlock the full potential of this data by combining it to generate powerful insights.

Get started with Health Connect...

Health & Fitness data types

See data types (https://developer.android.com/health-and-fitness/guides/health-connect/plan/data-types)

Health Connect stores and structures health and fitness data. It also accounts for how different data types are measured. These data types include heart rate measurements taken immediately, step count taken over time, and sleep data taken in sessions.

Measure skin temperature

(https://developer.android.com/health-and-fitness/guides/health-connect/develop/skin-temperature)

Health Connect provides a skin temperature data type to measure peripheral body temperature. This measurement is a particularly useful signal for detecting sleep quality, reproductive health, and the potential onset of illness.

Measure skin temperature...

Add exercise routes

(https://developer.android.com/health-and-fitness/guides/health-connect/develop/exercise-routes)

Exercise routes allow users to track a GPS route for associated exercise activities and share maps of their workouts with other apps.

Add exercise routes...

Track mindfulness

(https://developer.android.com/health-and-fitness/guides/health-connect/develop/mindfulness)

Health Connect provides a mindfulness data type to measure various aspects of mental health, such as stress and anxiety. Mindfulness is a data type that is part of overall wellness in Health Connect.

Track mindfulness...

Medical records

The Medical Records feature extends Health Connect to include basic medical data in the Fast Healthcare Interoperability Resources (FHIR®) format. It provides APIs for applications to read and write medical data, along with a user interface for browsing the data and managing permissions.

Learn more...

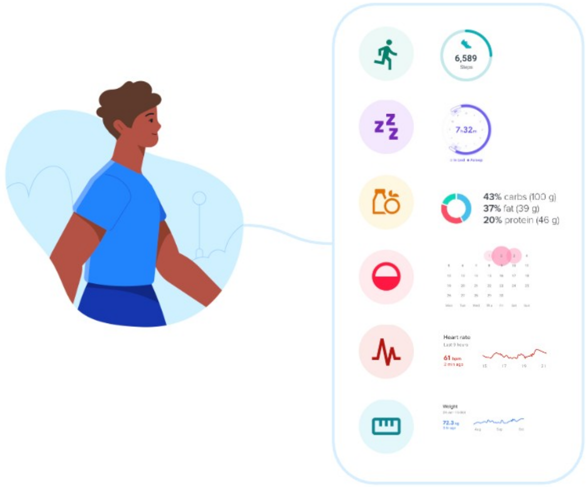

Functionalities

[illegible]ons on record and data synchronization

[illegible].com/health-and-fitness/guides/health-connect/[illegible]

[illegible] standard insert, update, and delete functions for record data [illegible]ality that allows client apps to synchronize data out of Health [illegible]

[illegible]ps://developer.android.com/health-and-fitness/guides/health-co[illegible]

FUNCTIONALITIES

Android compatibility

(https://developer.android.com/health-and-fitness/guides/health-connect/plan/availability)

Health Connect is compatible with Android SDK version 28 (Pie) and higher. The SDK [illegible] determine whether the Health Connect API is on the user's Android-powered device. [illegible] Connect triggers an availability check to determine whether the user's device is com[illegible]

Check compatibility (https://developer.android.com/health-and-fitness/guides/health-[illegible]

FUNCTIONALITIES

Reading with Health Connect

(https://developer.android.com/health-and-fitness/guides/health-connect/develop/read-data#reac[illegible]

Enable applications to continuously access a user's health data, even when the app is r[illegible] background. This ensures uninterrupted access for analysis, synchronization, and utiliz[illegible] seamless health tracking experience.

Read data (https://developer.android.com/health-and-fitness/guides/health-connect/devel[illegible]

Videos

Introducing new APIs f...

[Introducing new APIs for health and fitness in Health Connect by Android](https://www.youtube.com/watch?v=d14GVcnbTeo)

(https://www.youtube.com/watch?v=d14GVcnbTeo)

Learn about the new Health Connect APIs.

Watch...

Managing permission...

[Managing permissions in Health Connect](https://www.youtube.com/watch?v=C2I4OymO3S4)

(https://www.youtube.com/watch?v=C2I4OymO3S4)

Learn how to manager permissions in Health Connect.

Watch...

Reading and writing in ...

[Reading and writing in Health Connect](https://www.youtube.com/watch?v=NAx7Gv_Hk7E)

(https://www.youtube.com/watch?v=NAx7Gv_Hk7E)

Learn how to read and write data in Health Connect.

Watch...

Tips for a great Health...

[Tips for a great Health Connect integration](https://www.youtube.com/watch?v=yGAlBTTX9R4)

(https://www.youtube.com/watch?v=yGAlBTTX9R4)

Need help integrating Health Connect with your app? Watch this video to learn some tips.

Watch...

News

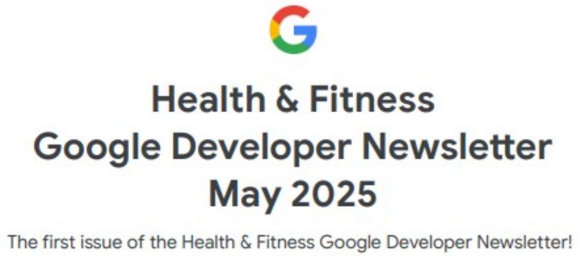

(https://developer.android.com/health-and-fitness/community/newsletters/2025/05)

Health & Fitness Google Developer Newsletter May 2025

(https://developer.android.com/health-and-fitness/community/newsletters/2025/05)

We shipped our first Health & Fitness Google Developer Newsletter! Learn about new features, updates, and community events in the newsletter.

Read more...

(https://android-developers.googleblog.com/2025/03/health-connect-jetpack-sdk-now-in-beta.html)

Unlock deeper health insights: Health Connect Jetpack SDK is now in beta and new feature updates

(https://android-developers.googleblog.com/2025/03/health-connect-jetpack-sdk-now-in-beta.html)

We're excited to announce significant Health Connect updates like the Jetpack SDK Beta, new datatypes and new permissions that will enable richer, more insightful app functionalities.

Learn more...

Health Connect Jetpack Library 1.1.0 is now

stable

We are excited to announce that the Health Connect Jetpack library has reached its 1.1.0 stable release. This milestone provides developers with the confidence and reliability needed to build production-ready health and fitness experiences at scale.

View release notes (https://developer.android.com/jetpack/androidx/releases/health-connect)

Connect with us (https://services.google.com/fb/forms/HealthDevNewsSignup)

Sign up for the Health & Fitness developer newsletter and get the latest news and updates straight to your inbox.

Sign up (https://services.google.com/fb/forms/HealthDevNewsSignup)

Content and code samples on this page are subject to the licenses described in the Content License (/license). Java and OpenJDK are trademarks or registered trademarks of Oracle and/or its affiliates.

Last updated 2025-11-10 UTC.
