# Supplementary material for: On the evolution of security and privacy in Android health APIs
Source: Front Digit Health. 2026 Oct 28;8:1818536. doi: 10.3389/fdgth.2026.1818536 (PMC13751995; doi:10.3389/fdgth.2026.1818536)
Supplement: Supplementary file 1 [file Datasheet1.zip › supplemental-data/documentation-sensors-api.pdf]

Starting in 2026, we'll be transitioning away from Google Fit APIs. For more information on the Google Fit migration, see the Migration Guide (/health-and-fitness/guides/health-connect/migrate/migration-guide).

Request background access to body sensor data

Android 13 and Wear OS 4 introduce a way for apps to access body sensors, such as heart rate, from the background. This new access model is similar to the one that introduced background location access in Android 10 (API level 29) (/training/location/permissions#request-background-location).

If your app needs to access body sensor information in background, such as when monitoring Health Services data in the background (/health-and-fitness/guides/health-services/monitor-background), you must request the `BODY_SENSORS_BACKGROUND` (/reference/android/Manifest.permission#BODY_SENSORS_BACKGROUND) permission.

Note: `BODY_SENSORS_BACKGROUND` is a restricted permission which cannot be held by your app until the installer adds your app to an allowlist, or until the user lets your app have the permission.

As described on the privacy best practices (/privacy/best-practices) page, apps should only ask for the `BODY_SENSORS_BACKGROUND` permission when it is critical to the user-facing feature, and they should properly disclose this to users.

The process for granting the permission depends on your app's target SDK version.

App targets Android 13 or higher

In addition to the existing `BODY_SENSORS` (/reference/android/Manifest.permission#BODY_SENSORS) permission, declare the `BODY_SENSORS_BACKGROUND` permission in your manifest file:

```
<uses-permission android:name="android.permission.BODY_SENSORS">
<uses-permission android:name="android.permission.BODY_SENSORS_BACKGROUND">
```

Then, your app must request (/training/permissions/requesting) the permissions in separate operations:

1. Check if `BODY_SENSORS` is granted. If not, request the permission.
2. Check if `BODY_SENSORS_BACKGROUND` is granted. If not, request the permission.

Caution: If your app requests both body sensor permissions at the same time, the system ignores the request and doesn't grant your app either permission.

On Android 13 (API level 33) and higher, the runtime permission dialog doesn't include the "Allow all the time" option. Instead, users must enable all-the-time background sensor access from system settings, as shown in figure 1. When you request the `BODY_SENSORS_BACKGROUND` permission after granting the `BODY_SENSORS` permission, you can help users navigate to this settings page. If users decline all-the-time access, they should be able to continue using your app.

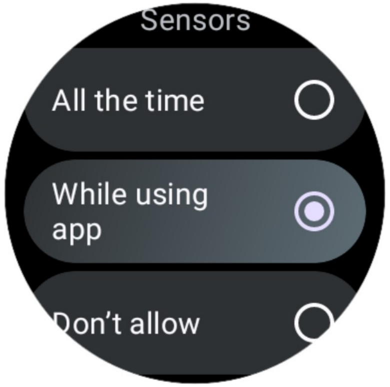

Figure 1. *Sensors setting includes an option called* ***All the time****, which grants background sensor data*

access.

App targets an earlier version

When your app targets a version of Android earlier than Android 13, background access isn't granted automatically when you request the `BODY_SENSORS` permission. Instead, users see a system dialog that invites users to navigate to your app's sensor permission settings, as shown in figure 2. Then, users must enable background sensor usage on that settings page.

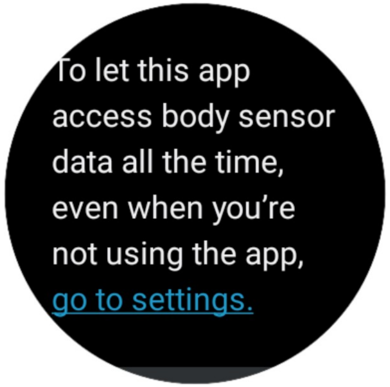

Figure 2. *Permission dialog includes a link to navigate users to the app's sensor permissions in system settings.*

Users can decline the background access. It has the same effect as revoking the `BODY_SENSORS` permission while your app is running in the background. When an app is using `PassiveMonitoringClient` (/reference/kotlin/androidx/health/services/client/PassiveMonitoringClient) without background access permission and goes into the background, the app loses the `BODY_SENSORS` permission, and the `onPermissionLost()` (/reference/kotlin/androidx/health/services/client/PassiveListenerService#onPermissionLost()) callback is called. For these reasons, it's especially important that you follow best practices for requesting runtime permissions.

Content and code samples on this page are subject to the licenses described in the Content License (/license). Java and OpenJDK are trademarks or registered trademarks of Oracle and/or its affiliates.

Last updated 2025-11-10 UTC.
