# Supplementary material for: On the evolution of security and privacy in Android health APIs
Source: Front Digit Health. 2026 Oct 28;8:1818536. doi: 10.3389/fdgth.2026.1818536 (PMC13751995; doi:10.3389/fdgth.2026.1818536)
Supplement: Supplementary file 1 [file Datasheet1.zip › supplemental-data/empirical-tests-matrix-1.pdf]

A Empirical OWASP MASTG Verification Matrix

Table 1: Detailed Technical Breakdown of OWASP MASTG Tests Executed on Health Connect.

Test ID	Tested Component	Input Data	Test Procedure	Evidence	Result
**TEST-0200** *(MASVS-STORAGE-2)*	Shared external storage directory: `/storage/emulated/0` Package: `com.google.android.apps.healthdata`	Clean installation of Health Connect from Google Play Store; dynamic execution navigating permissions, data categories, and source priority settings.	1. Create a virtual machine snapshot to preserve laboratory baseline.<br>2. Uninstall Health Connect to establish an unpolluted filesystem state.<br>3. Generate an initial sorted file listing of `/storage/emulated/0` via ADB into `before_HC.txt`.<br>4. Install Health Connect and systematically exercise all core UI screens.<br>5. Generate a post-execution sorted file listing of external storage into `after_HC.txt`.<br>6. Compare listings to capture newly created files in `diff.txt`.	Files generated: `before_HC.txt` (12 files), `after_HC.txt` (12 files), and `diff.txt` (0 bytes). No unencrypted or sensitive files were created in external storage. Figure 1.	**PASSED**
**TEST-0202** *(MASVS-STORAGE-2)*	`AndroidManifest.xml` of the `.apk` and decompiled Java classes: `defpackage/ejr.java`, `defpackage/bio.java`, `defpackage/wi.java`, `defpackage/bed.java`, `defpackage/bec.java`	Extracted `.apk` of Health Connect (build `2026.02.19.00.release`, `versionCode=242770`, `targetSdkVersion=36`).	1. Inspect declared permissions with `aapt2 dump permissions` filtering for shared/external storage flags.<br>2. Decompile the APK using JADX.<br>3. Execute regex searches across sources for external storage APIs (`MediaStore`, `getExternalFilesDir`, etc.).<br>4. Perform manual source code audit on identified occurrences and `MediaStore` calls.<br>5. Perform a targeted search for MediaStore write patterns (`ContentResolver.insert`, `openOutputStream`, `IS_PENDING`).	no external storage permissions found.<br>`MediaStore` is used strictly for read/query operations.<br>Figure 2.	**PASSED**

Continued on next page

Table 1: Detailed Technical Breakdown of Selected OWASP MASTG Tests Executed on Health Connect (Continued)

Test ID	Tested Component	Input Data	Test Procedure	Evidence	Result
**TEST-0207** *(MASVS-STORAGE-1)*	Private application sandbox directory: `/data/user/0/com.google.android.apps.healthdata/` Database: `databases/healthcore.db` Tables: `IntervalData`, `SampleData`, `SeriesData`, `ApplicationInfo`	Synthetic health records populated via Health Connect Toolbox v2.3.3: 10,000 steps (`count`), 80 kg body weight (`weight`), and heart rate (`bpm`).	1. Force-stop Health Connect and pull baseline private sandbox via root ADB into `before/`.<br>2. Insert synthetic health records using Health Connect Toolbox.<br>3. Force-stop Health Connect and the client tool, then pull updated sandbox into `after/`.<br>4. Compare directories and file sizes via `diff -u` to isolate active database and WAL journal files.<br>5. Inspect `healthcore.db` schema and row count deltas using Python `sqlite3` scripts.<br>6. Execute a Python decoder over binary `content_values` BLOBs in new rows to extract ASCII strings and decode varints and doubles.	`files_diff.txt` revealed active SQLite WAL journals (`healthcore.db-wal`). `db_counts_compare.txt` showed row increases across `SampleData`, `SeriesData`, and `IntervalData`. `decoded_new_values_after.txt` recovered string `count` and exact integer `10000` (decoded via varint), as well as IEEE double `80.08` for `weight`, directly from database BLOBs without cryptographic keys. Figure 3, 4, 5, 6 and 7.	**FAIL**
**TEST-0254** *(MASVS-PRIVACY-1)*	`AndroidManifest.xml` of the `.apk`	Application `uses-permission` tags from `AndroidManifest.xml` and platform dangerous permissions list from Android 13 (API 33).	1. Dump all declared permissions from `base.apk` using `aapt2 dump permissions`.<br>2. Extract and sort unique `uses-permission` declarations into `permisos_healthconnect.txt`.<br>3. Retrieve OS dangerous permissions from device using `adb shell pm list permissions -g -d` into `permisos_dangerous_android.txt`.<br>4. Intersect both lists using `comm -12` to isolate declared dangerous permissions.<br>5. Analyse the dangerous permissions requested by the application according to its context.	`android.permission.POST_NOTIFICATIONS`. was the only dangerous permission requested. No sensor, location, or storage permissions were requested.<br>The permission is deemed trustworthy within the Health Connect context and a subsequent text audit (TEST-0315) confirmed no sensitive health data is leaked. Figure 8.	**PASSED**

Continued on next page

Table 1: Detailed Technical Breakdown of Selected OWASP MASTG Tests Executed on Health Connect (Continued)

Test ID	Tested Component	Input Data	Test Procedure	Evidence	Result
**TEST-0262** *(MASVS-STORAGE-2)*	`AndroidManifest.xml` and compiled backup rule resources: `res/Qq.xml` (logical `xml/backup_rules`) and `res/ib.xml` (logical `xml/backup_rules_legacy`)	Application backup attributes (`allowBackup`, `dataExtractionRules`, `fullBackupContent`) and XML configuration trees inside `base.apk`.	1. Extract package SDK baseline (`minSdkVersion=32`, `targetSdkVersion=36`) via `aapt2 dump badging`.<br>2. Extract manifest backup attributes from `AndroidManifest_base_xmltree.txt`.<br>3. Resolve resource IDs (`0x7f170000`, `0x7f170001`) to XML aliases using `aapt2 dump resources`.<br>4. Iterate over compiled XML files using `aapt2 dump xmltree --file` to pinpoint physical paths of rules files.<br>5. Audit inclusion/exclusion rules for `cloud-backup` and `device-transfer` transport channels.	Customized backup agents and rule mappings are confirmed at resource IDs (`0x7f170000`, `0x7f170001`). They confirmed that cloud-backup excludes the root directory, while device-transfer explicitly includes `healthcore.db`. Although sensitive information is included in device-to-device transfers, it is strictly limited to health-related data, which constitutes the core purpose of Health Connect. Since no other sensitive data was found, the test is considered passed. Figure 9.	**PASSED**
**TEST-0315** *(MASVS-PLATFORM-3)*	Notification dispatchers and resources: `defpackage/cgc.java` (`export_data`), `defpackage/cdy.java` (`delete_data`), `defpackage/cxl.java` (`unused_apps`), `res/values/strings.xml`, `res/values/plurals.xml`	Decompiled Java source files and compiled XML string/plural resource pools of `base.apk`.	1. Verify `POST_NOTIFICATIONS` permission declaration and SDK level requirements.<br>2. Decompile `base.apk` into Java sources using JADX.<br>3. Search source tree with `grep` for notification APIs, channels, and manager builders.<br>4. Filter candidate classes and isolate active notification dispatch routines.<br>5. Extract `R.string.*` and `R.plurals.*` resource identifiers used in notification builders.<br>6. Resolve string IDs against `strings.xml` and `plurals.xml` to audit visible notification copy.	export, delete and unused-app notification channels were discovered.<br>Resolved strings confirmed all notification messages contain only generic status indicators (e.g., "Deleting Health Connect data", "Exporting Health Connect data") and exclude user health metrics, timestamps, medical values, and personal identifiers. Figure 10.	**PASSED**

```
uru@uru:~/UVa/TFG/Pruebas/test200$ adb shell dumpsys package com.google.android.apps.healthdata | gr
ep -E "versionName|versionCode"
    versionCode=253292 minSdk=32 targetSdk=36
    versionName=2026.04.16.00.release
uru@uru:~/UVa/TFG/Pruebas/test200$ adb shell find /storage/emulated/0 -type f 2>/dev/null | sort > a
fter_HC.txt
uru@uru:~/UVa/TFG/Pruebas/test200$ comm -13 before_HC.txt after_HC.txt > diff.txt
uru@uru:~/UVa/TFG/Pruebas/test200$ cat diff.txt
uru@uru:~/UVa/TFG/Pruebas/test200$ wc -l before_HC.txt after_HC.txt
  12 before_HC.txt
  12 after_HC.txt
  24 total
```

Figure 1: Execution of TEST-0200.

```
uru@uru:~/UVa/TFG/Pruebas/test202$ aapt2 dump permissions ../base.apk | grep -Ei 'EXTERNAL_STORAGE|M
ANAGE_EXTERNAL_STORAGE|READ_MEDIA_|ACCESS_MEDIA_LOCATION' | tee permisos_almacenamiento.txt
uru@uru:~/UVa/TFG/Pruebas/test202$ wc -c permisos_almacenamiento.txt
0 permisos_almacenamiento.txt
uru@uru:~/UVa/TFG/Pruebas/test202$ grep -RInE 'getExternal(StoragePublicDirectory|StorageDirectory|F
ilesDir|CacheDir|MediaDirs|FilesDirs)|MediaStore|Environment\.DIRECTORY_|ContentResolver\.insert|ope
nOutputStream|RELATIVE_PATH|IS_PENDING' ../test315/healthconnect\_jadx | tee referencias_almacenamie
nto_externo.txt
../test315/healthconnect_jadx/sources/defpackage/bio.java:7:import android.provider.MediaStore;
../test315/healthconnect_jadx/sources/defpackage/bio.java:106:                        requireOrigina
l = MediaStore.setRequireOriginal(requireOriginal);
../test315/healthconnect_jadx/sources/defpackage/wi.java:10:import android.provider.MediaStore;
../test315/healthconnect_jadx/sources/defpackage/wi.java:491:        return MediaStore.openAssetFile
Descriptor(contentResolver, uri, "r", null);
../test315/healthconnect_jadx/sources/defpackage/ejr.java:55:            File externalFilesDir = con
text.getExternalFilesDir(null);
../test315/healthconnect_jadx/sources/defpackage/bed.java:6:import android.provider.MediaStore;
../test315/healthconnect_jadx/sources/defpackage/bed.java:21:        return this.b.query(MediaStore.
Video.Thumbnails.EXTERNAL_CONTENT_URI, a, "kind = 1 AND video_id = ?", new String[]{lastPathSegment}
, null);
../test315/healthconnect_jadx/sources/defpackage/bec.java:6:import android.provider.MediaStore;
../test315/healthconnect_jadx/sources/defpackage/bec.java:21:        return this.b.query(MediaStore.
Images.Thumbnails.EXTERNAL_CONTENT_URI, a, "kind = 1 AND image_id = ?", new String[]{lastPathSegment
}, null);
uru@uru:~/UVa/TFG/Pruebas/test202$ sed -E 's#^\.\./test315/healthconnect_jadx/sources/##' referencia
s_almacenamiento_externo.txt
defpackage/bio.java:7:import android.provider.MediaStore;
defpackage/bio.java:106:                        requireOriginal = MediaStore.setRequireOriginal(requ
ireOriginal);
defpackage/wi.java:10:import android.provider.MediaStore;
defpackage/wi.java:491:        return MediaStore.openAssetFileDescriptor(contentResolver, uri, "r",
null);
defpackage/ejr.java:55:            File externalFilesDir = context.getExternalFilesDir(null);
defpackage/bed.java:6:import android.provider.MediaStore;
defpackage/bed.java:21:        return this.b.query(MediaStore.Video.Thumbnails.EXTERNAL_CONTENT_URI,
 a, "kind = 1 AND video_id = ?", new String[]{lastPathSegment}, null);
defpackage/bec.java:6:import android.provider.MediaStore;
defpackage/bec.java:21:        return this.b.query(MediaStore.Images.Thumbnails.EXTERNAL_CONTENT_URI
, a, "kind = 1 AND image_id = ?", new String[]{lastPathSegment}, null);
uru@uru:~/UVa/TFG/Pruebas/test202$ grep -RInE "ContentResolver\.insert\(|openOutputStream\(|RELATIVE
_PATH|IS_PENDING" ../test315/healthconnect_jadx | tee referencias_mediastore_escritura.txt
uru@uru:~/UVa/TFG/Pruebas/test202$ wc -c referencias_mediastore_escritura.txt
0 referencias_mediastore_escritura.txt
```

Figure 2: Execution of TEST-0202.

```
uru@uru:~/UVa/TFG/Pruebas/test207$ adb shell am force-stop com.google.android.apps.healthdata
uru@uru:~/UVa/TFG/Pruebas/test207$ adb pull /data/user/0/com.google.android.apps.healthdata before/
/data/user/0/com.google.android.apps.healthdata/... files pulled. 3.3 MB/s (365681 bytes in 0.105s)
uru@uru:~/UVa/TFG/Pruebas/test207$ find before/com.google.android.apps.healthdata -type f | sed 's#b
efore/com.google.android.apps.healthdata/##' | sort | tee before_files.txt
cache/data/user/0/com.google.android.apps.healthdata/no_backup/androidx.work.workdb.lck
databases/healthcore.db
files/AccountSyncData.pb
files/OnboardingStatus.pb
files/PermissionRequestCounterData.pb
files/PrioritySyncData.pb
files/RateLimiterProtoDataStore.pb
files/SessionAdoptionStatusDataStore.pb
files/tiktok/103243289
no_backup/androidx.work.workdb
no_backup/androidx.work.workdb-shm
no_backup/androidx.work.workdb-wal
shared_prefs/primes.xml
uru@uru:~/UVa/TFG/Pruebas/test207$ adb shell am force-stop androidx.health.connect.client.devtool
uru@uru:~/UVa/TFG/Pruebas/test207$ adb shell am force-stop com.google.android.apps.healthdata
uru@uru:~/UVa/TFG/Pruebas/test207$ adb pull /data/user/0/com.google.android.apps.healthdata after/
/data/user/0/com.google.android.apps.healthdata/... files pulled. 2.3 MB/s (588003 bytes in 0.246s)
uru@uru:~/UVa/TFG/Pruebas/test207$ find after/com.google.android.apps.healthdata -type f | sed 's#af
ter/com.google.android.apps.healthdata/##' | sort | tee after_files.txt
cache/data/user/0/com.google.android.apps.healthdata/no_backup/androidx.work.workdb.lck
databases/healthcore.db
databases/healthcore.db-shm
databases/healthcore.db-wal
files/AccountSyncData.pb
files/OnboardingStatus.pb
files/PermissionRequestCounterData.pb
files/PrioritySyncData.pb
files/RateLimiterProtoDataStore.pb
files/SessionAdoptionStatusDataStore.pb
files/tiktok/103243289
no_backup/androidx.work.workdb
no_backup/androidx.work.workdb-shm
no_backup/androidx.work.workdb-wal
shared_prefs/primes.xml
uru@uru:~/UVa/TFG/Pruebas/test207$ diff -u before_files.txt after_files.txt | tee files_diff.txt
--- before_files.txt    2026-05-31 21:12:34.178915094 +0200
+++ after_files.txt     2026-05-31 21:20:44.651174055 +0200
@@ -1,5 +1,7 @@
 cache/data/user/0/com.google.android.apps.healthdata/no_backup/androidx.work.workdb.lck
 databases/healthcore.db
+databases/healthcore.db-shm
+databases/healthcore.db-wal
 files/AccountSyncData.pb
 files/OnboardingStatus.pb
 files/PermissionRequestCounterData.pb
```

Figure 3: Execution of TEST-0207 (1).

```
uru@uru:~/UVa/TFG/Pruebas/test207$ find before/com.google.android.apps.healthdata -type f -printf '%
P %s\n' | sort > before_sizes.txt
uru@uru:~/UVa/TFG/Pruebas/test207$ find after/com.google.android.apps.healthdata -type f -printf '%P
 %s\n' | sort > after_sizes.txt
uru@uru:~/UVa/TFG/Pruebas/test207$ diff -u before_sizes.txt after_sizes.txt | tee files_size_diff.tx
t
--- before_sizes.txt    2026-05-31 21:22:28.025759064 +0200
+++ after_sizes.txt     2026-05-31 21:22:36.071961213 +0200
@@ -1,10 +1,12 @@
 cache/data/user/0/com.google.android.apps.healthdata/no_backup/androidx.work.workdb.lck 0
 databases/healthcore.db 192512
+databases/healthcore.db-shm 32768
+databases/healthcore.db-wal 189552
 files/AccountSyncData.pb 11
 files/OnboardingStatus.pb 2
 files/PermissionRequestCounterData.pb 7803
 files/PrioritySyncData.pb 2
-files/RateLimiterProtoDataStore.pb 139
+files/RateLimiterProtoDataStore.pb 141
 files/SessionAdoptionStatusDataStore.pb 2
 files/tiktok/103243289 4
 no_backup/androidx.work.workdb 4096
uru@uru:~/UVa/TFG/Pruebas/test207$ file before/com.google.android.apps.healthdata/databases/healthco
re.db after/com.google.android.apps.healthdata/databases/healthcore.db after/com.google.android.apps
.healthdata/databases/healthcore.db-wal | tee db_file_type.txt
before/com.google.android.apps.healthdata/databases/healthcore.db:    SQLite 3.x database, user vers
ion 22, last written using SQLite version 3032002, writer version 2, read version 2, file counter 31
, database pages 47, 1st free page 40, free pages 4, cookie 0x27, schema 4, largest root page 37, UT
F-8, vacuum mode 1, version-valid-for 31
after/com.google.android.apps.healthdata/databases/healthcore.db:     SQLite 3.x database, user vers
ion 22, last written using SQLite version 3032002, writer version 2, read version 2, file counter 31
, database pages 47, 1st free page 40, free pages 4, cookie 0x27, schema 4, largest root page 37, UT
F-8, vacuum mode 1, version-valid-for 31
after/com.google.android.apps.healthdata/databases/healthcore.db-wal: SQLite Write-Ahead Log, versio
n 3007000
```

Figure 4: Execution of TEST-0207 (2).

```
=== before ===
SampleData: 5
SeriesData: 2
IntervalData: 7
DataAccessLog: 61
ApplicationInfo: 1

=== after ===
SampleData: 6
SeriesData: 4
IntervalData: 8
DataAccessLog: 65
ApplicationInfo: 1
```

Figure 5: Execution of TEST-0207 (3).

```
(1, 'androidx.health.connect.client.devtool', 'Health Connect Toolbox')
```

Figure 6: Execution of TEST-0207 (4).

```
Tabla: SampleData
row_id: 6
data_type_id: 53
cadenas_ascii: ['weight']
valor_double_detectado: 5.591219485744295e+252
valor_double_detectado: 0.08

Tabla: SeriesData
row_id: 3
data_type_id: 56
cadenas_ascii: ['bpm', 'bpm', 'bpm', 'bpm', 'bpm', 'bpm', 'bpm', 'bpm', 'bpm', 'bpm']
valores_bpm_detectados: [57, 79, 61, 66, 66, 62, 74, 74, 56, 68]

Tabla: SeriesData
row_id: 4
data_type_id: 56
cadenas_ascii: ['bpm', 'bpm', 'bpm', 'bpm', 'bpm', 'bpm', 'bpm', 'bpm', 'bpm', 'bpm']
valores_bpm_detectados: [63, 53, 80, 59, 64, 72, 75, 59, 53, 54]

Tabla: IntervalData
row_id: 8
data_type_id: 13
cadenas_ascii: ['count']
valor_count_detectado: 10000
```

Figure 7: Execution of TEST-0207 (5).

```
uru@uru:~/UVa/TFG/Pruebas/test254$ aapt2 dump permissions ../base.apk | tee evidencia_permisos_HC.tx
t
package: com.google.android.apps.healthdata
uses-permission: name='android.permission.WAKE_LOCK'
uses-permission: name='android.permission.RECEIVE_BOOT_COMPLETED'
uses-permission: name='com.google.android.providers.gsf.permission.READ_GSERVICES'
uses-permission: name='android.permission.ACCESS_NETWORK_STATE'
uses-permission: name='android.permission.GET_PACKAGE_SIZE'
uses-permission: name='android.permission.FOREGROUND_SERVICE'
uses-permission: name='android.permission.MIGRATE_HEALTH_CONNECT_DATA'
uses-permission: name='android.permission.PACKAGE_USAGE_STATS'
uses-permission: name='android.permission.POST_NOTIFICATIONS'
uses-permission: name='android.permission.REQUEST_DELETE_PACKAGES'
uses-permission: name='android.permission.INTERNET'
permission: com.google.android.apps.healthdata.permission.START_ONBOARDING
uses-permission: name='com.google.android.apps.healthdata.permission.START_ONBOARDING'
permission: com.google.android.apps.healthdata.DYNAMIC_RECEIVER_NOT_EXPORTED_PERMISSION
uru@uru:~/UVa/TFG/Pruebas/test254$ grep "^uses-permission" evidencia_permisos_HC.txt | sed -E "s/.*n
ame='([^']+)'.*/\1/" | sort -u | tee permisos_healthconnect.txt
android.permission.ACCESS_NETWORK_STATE
android.permission.FOREGROUND_SERVICE
android.permission.GET_PACKAGE_SIZE
android.permission.INTERNET
android.permission.MIGRATE_HEALTH_CONNECT_DATA
android.permission.PACKAGE_USAGE_STATS
android.permission.POST_NOTIFICATIONS
android.permission.RECEIVE_BOOT_COMPLETED
android.permission.REQUEST_DELETE_PACKAGES
android.permission.WAKE_LOCK
com.google.android.apps.healthdata.permission.START_ONBOARDING
com.google.android.providers.gsf.permission.READ_GSERVICES
uru@uru:~/UVa/TFG/Pruebas/test254$ adb shell pm list permissions -g -d | grep "permission:" | sed 's
/.*permission://' | tr -d '\r' | sort -u > permisos_dangerous_android.txt
uru@uru:~/UVa/TFG/Pruebas/test254$ comm -12 permisos_healthconnect.txt permisos_dangerous_android.tx
t | tee permisos_dangerous_detectados.txt
android.permission.POST_NOTIFICATIONS
```

Figure 8: Execution of TEST-0254.

```
uru@uru:~/UVa/TFG/Pruebas/test262$ while read -r f; do
  out=$(aapt2 dump xmltree --file "$f" ../base.apk 2>/dev/null)
  if echo "$out" | grep -qiE "data-extraction-rules|full-backup-content"; then
    echo "===== $f ====="
    echo "$out"
  fi
done < todos_los_xml.txt \
| tee reglas_backup_principales.txt
===== res/Qq.xml =====
N: android=http://schemas.android.com/apk/res/android (line=2)
  E: data-extraction-rules (line=2)
      E: cloud-backup (line=3)
        A: disableIfNoEncryptionCapabilities=true
          E: exclude (line=4)
            A: domain="root" (Raw: "root")
            A: path="." (Raw: ".")
      E: device-transfer (line=6)
          E: include (line=7)
            A: domain="database" (Raw: "database")
            A: path="healthcore.db" (Raw: "healthcore.db")
          E: include (line=8)
            A: domain="file" (Raw: "file")
            A: path="OnboardingStatus.pb" (Raw: "OnboardingStatus.pb")
          E: include (line=9)
            A: domain="file" (Raw: "file")
            A: path="AutoDeleteProto.pb" (Raw: "AutoDeleteProto.pb")
===== res/ib.xml =====
E: full-backup-content (line=2)
    E: include (line=3)
      A: domain="database" (Raw: "database")
      A: path="healthcore.db" (Raw: "healthcore.db")
      A: requireFlags="deviceToDeviceTransfer" (Raw: "deviceToDeviceTransfer")
    E: include (line=4)
      A: domain="file" (Raw: "file")
      A: path="OnboardingStatus.pb" (Raw: "OnboardingStatus.pb")
      A: requireFlags="deviceToDeviceTransfer" (Raw: "deviceToDeviceTransfer")
    E: include (line=6)
      A: domain="file" (Raw: "file")
      A: path="AutoDeleteProto.pb" (Raw: "AutoDeleteProto.pb")
      A: requireFlags="deviceToDeviceTransfer" (Raw: "deviceToDeviceTransfer")
```

Figure 9: Execution of TEST-0262.

```
uru@uru:~/UVa/TFG/Pruebas/test315$ jadx -d healthconnect_jadx ../base.apk 2>&1 | tee jadx_decompilat
ion.log
INFO  - loading ...
INFO  - processing ...
ERROR - finished with errors, count: 7
uru@uru:~/UVa/TFG/Pruebas/test315$ ls healthconnect_jadx
resources  sources
uru@uru:~/UVa/TFG/Pruebas/test315$ find healthconnect_jadx/sources -type f -name "*.java" | wc -l
7357
uru@uru:~/UVa/TFG/Pruebas/test315$ grep -RIn -E "NotificationManager|NotificationChannel|createNotif
icationChannel|\.notify\(|NotificationCompat|Notification\.Builder|setContentTitle|setContentText|ex
port_data|delete_data|unused_apps_notification|DataNotificationManager" healthconnect_jadx/sources >
 notification_api_refs.txt
uru@uru:~/UVa/TFG/Pruebas/test315$ cut -d: -f1 notification_api_refs.txt | sort -u | tee notificatio
n_candidate_files.txt
healthconnect_jadx/sources/androidx/work/impl/foreground/SystemForegroundService.java
healthconnect_jadx/sources/com/google/android/apps/healthdata/autodelete/ui/impl/AutoDeleteFragment.
java
healthconnect_jadx/sources/com/google/android/apps/healthdata/managedata/ExportDataView.java
healthconnect_jadx/sources/com/google/android/apps/healthdata/R.java
healthconnect_jadx/sources/defpackage/aor.java
healthconnect_jadx/sources/defpackage/bql.java
healthconnect_jadx/sources/defpackage/cbk.java
healthconnect_jadx/sources/defpackage/cdy.java
healthconnect_jadx/sources/defpackage/cfv.java
healthconnect_jadx/sources/defpackage/cgc.java
healthconnect_jadx/sources/defpackage/ckt.java
healthconnect_jadx/sources/defpackage/cwm.java
healthconnect_jadx/sources/defpackage/cxl.java
healthconnect_jadx/sources/defpackage/dtp.java
healthconnect_jadx/sources/defpackage/gfu.java
healthconnect_jadx/sources/defpackage/sk.java
healthconnect_jadx/sources/defpackage/yr.java
```

Figure 10: Execution of TEST-0315.
