# Supplementary material for: On the evolution of security and privacy in Android health APIs
Source: Front Digit Health. 2026 Oct 28;8:1818536. doi: 10.3389/fdgth.2026.1818536 (PMC13751995; doi:10.3389/fdgth.2026.1818536)
Supplement: Supplementary file 1 [file Datasheet1.zip › supplemental-data/traceability-matrix.pdf]

A Traceability Matrix: Threat Model to Verification

Table 1: Traceability Matrix mapping Threat Model Claims to Verification. **Abbreviations:** HC = Health Connect, GF = Google Fit, API = Android Sensor API.

**Threat Model (Claim)**				**Results & Evidence**		
**Assets**	**Adversaries**	**Comparative Criteria (Requirement)**	**Technical Measures (API Feature)**	**Result**	**Evidences**	**Verification (*Direct/Indirect*)**
Health data stored on the device & Metadata	Network-level eavesdroppers, Unauthorised third parties	Confidentiality (Security)	Storage Encryption, Storage and OS Interaction, Data Granularity and Permissions	HC: Partial<br>GF: Not supported<br>API: Dev-dependent	HC: [1, 2], [1], [2], [3]<br>GF: [3], [4], [5]<br>API: [4], [6]	HC: OWASP TEST-0207 [*Direct*: plaintext SQLite extraction] (FAIL)
Health data stored on the device & Metadata	Network-level eavesdroppers, Unauthorised third parties	Integrity (Security)	Storage and OS Interaction, Operational Model	HC: Native<br>GF: Not supported<br>API: Dev-dependent	HC: [1, 2], [7]<br>GF: [3], [4]<br>API: [4], [8]	HC: OWASP TEST-0200, TEST-0202 [*Indirect*: sandbox isolation prevents API bypass] (PASSED)
Health data stored on the device & Metadata	Network-level eavesdroppers, Unauthorised third parties	Availability (Security)	Operational Model	HC: Native<br>GF: Not supported<br>API: Native	HC: [2], [1], [3]<br>GF: [3], [4]<br>API: [4], [8]	HC: Architectural verification [*Direct*: Local operational model with no external network or cloud dependencies] (PASSED)

Continued on next page

[1] https://developer.android.com/codelabs/health-connect#0
[2] https://android.googlesource.com/platform//frameworks/support/+/refs/heads/androidx-health-connect-release/health/connect/connect-client/src/main/java/androidx/health/connect/client/HealthConnectClient.kt
[3] https://android.googlesource.com/platform//frameworks/support/+/refs/heads/androidx-health-connect-release/health/connect/connect-client/
[4] https://developers.google.com/fit/android
[5] https://developers.google.com/fit/android/authorization
[6] https://developer.android.com/reference/android/Manifest.permission#BODY_SENSORS
[7] https://developer.android.com/health-and-fitness/health-connect/metadata?hl=en
[8] https://android.googlesource.com/platform/frameworks/base/+/refs/heads/master/core/java/android/hardware/SensorManager.java

Table 1: Traceability Matrix mapping Threat Model Claims to Verification (Continued)

Threat Model (Claim)				Results & Evidence		
**Assets**	**Adversaries**	**Comparative Criteria (Requirement)**	**Technical Measures (API Feature)**	**Result**	**Evidences**	**Verification**
Metadata (origin, sources)	Network-level eavesdroppers, Unauthorised third parties	Authenticity (Security)	Storage and OS Interaction, Data Granularity and Permissions	HC: Native<br>GF: Partial<br>API: Native	HC: [1], [7]<br>GF: [3], [9]<br>API: [4]	HC: OWASP TEST-0200, TEST-0202 [*Indirect*: sandbox isolation prevents API bypass] + Architectural verification [*Direct*: OS-mediated IPC caller UID & origin metadata; see Sec. 3.2.3](PASSED)
Health data stored on the device	Unauthorised third parties	Data Minimisation (Privacy)	Data Granularity and Permissions	HC: Native<br>GF: Not supported<br>API: Not supported	HC: [2], [3]<br>GF: [3], [5]<br>API: [4], [6]	HC: OWASP TEST-0254, TEST-0315 [*Direct*: least-privilege declaration and notification payload minimisation] (PASSED)
Health data stored on the device	Unauthorised third parties	User's Ability to Manage Consent (Privacy)	Data Granularity and Permissions, Operational Model	HC: Native<br>GF: Partial<br>API: Dev-dependent	HC: [2], [3]<br>GF: [3], [4], [5]<br>API: [4], [6]	HC: OWASP TEST-0254 [*Indirect*: No undeclared dangerous permissions] & OWASP TEST-0262 [*Indirect*: prevents unconsented remote persistence] (PASSED)
Health data stored on the device	Unauthorised third parties	Transparency (Privacy)	Data Granularity and Permissions	HC: Native<br>GF: Not supported<br>API: Not supported	HC: [1, 2], [2], [3]<br>GF: [3], [5]<br>API: [4], [6]	HC: OWASP TEST-0254, TEST-0315 [*Indirect*: technical visibility & transparent user messaging] (PASSED)

Continued on next page

[9] https://developers.google.com/fit/android/data-attribution?hl=en

Table 1: Traceability Matrix mapping Threat Model Claims to Verification (Continued)

**Threat Model (Claim)**				**Results & Evidence**		
**Assets**	**Adversaries**	**Comparative Criteria (Requirement)**	**Technical Measures (API Feature)**	**Result**	**Evidences**	**Verification**
Health data stored on the device & Metadata	Unauthorised third parties	Accuracy (Privacy)	Storage and OS Interaction, Operational Model	HC: Native<br>GF: Partial<br>API: Native	HC: [1, 2], [7]<br>GF: [3], [9]<br>API: [4]	HC: Architectural verification [*Direct*: origin metadata attribution & record update/rectification APIs; see Sec. 3.2.3] (PASSED)
Health data stored on the device	Unauthorised third parties	Storage Limitation (Privacy)	Storage and OS Interaction, Operational Model	HC: Native<br>GF: Partial<br>API: Dev-dependent	HC: [1, 2], [1]<br>GF: [3], [9]<br>API: [4]	HC: OWASP TEST-0262 [*Indirect*: backup rules prevent persistent remote cloud retention] (PASSED)

References

[1] Mayrhofer R, Stoep JV, Brubaker C, Hackborn D, Bonné B, Tuncay GS, et al. The Android Platform Security Model. *arXiv preprint arXiv:2503.02829* (2023). `https://arxiv.org/abs/2503.02829`.

[2] Google. Health Connect: simplify connectivity between apps. *Online Resource* (2024). `https://developer.android.com/health-and-fitness/guides/health-connect` (Last accessed: 15/09/2026).

[3] Google. Android APIs — Google Fit. *Online Resource* (2024). `https://developers.google.com/fit/android?hl=en` (Last accessed: 15/09/2026).

[4] Google. Request background access to body sensor data. *Online Resource* (2024). `https://developer.android.com/health-and-fitness/guides/health-services/background-body-sensors` (Last accessed: 15/09/2026).
